# Multifunctional oxidosqualene cyclases and cytochrome P450 involved in the biosynthesis of apple fruit triterpenic acids

**DOI:** 10.1111/nph.13996

**Published:** 2016-05-23

**Authors:** Christelle M. Andre, Sylvain Legay, Amélie Deleruelle, Niels Nieuwenhuizen, Matthew Punter, Cyril Brendolise, Janine M. Cooney, Marc Lateur, Jean‐François Hausman, Yvan Larondelle, William A. Laing

**Affiliations:** ^1^ Department of Environmental Research and InnovationLuxembourg Institute of Science and TechnologyAvenue des Hauts‐FourneauxL‐4362Esch/AlzetteLuxembourg; ^2^ Institut des Sciences de la VieUCLouvainB‐1348Louvain‐la‐NeuveBelgium; ^3^ The New Zealand Institute for Plant & Food Research LimitedMt Albert Research CentrePrivate Bag 92 169Auckland1142New Zealand; ^4^ The New Zealand Institute for Plant & Food Research LimitedRuakuraHamilton3240New Zealand; ^5^ Walloon Agricultural Research CentreRue de LirouxB‐5030GemblouxBelgium

**Keywords:** apple, betulinic acid, cytochrome P450, germanicol, *Malus* × *domestica*, oxydosqualene cyclase, triterpene, ursolic acid

## Abstract

- Apple (*Malus *× *domestica*) accumulates bioactive ursane‐, oleanane‐, and lupane‐type triterpenes in its fruit cuticle, but their biosynthetic pathway is still poorly understood.
- We used a homology‐based approach to identify and functionally characterize two new oxidosqualene cyclases (MdOSC4 and MdOSC5) and one cytochrome P450 (CYP716A175). The gene expression patterns of these enzymes and of previously described oxidosqualene cyclases were further studied in 20 apple cultivars with contrasting triterpene profiles.
- *MdOSC4* encodes a multifunctional oxidosqualene cyclase producing an oleanane‐type triterpene, putatively identified as germanicol, as well as β‐amyrin and lupeol, in the proportion 82 : 14 : 4. MdOSC5 cyclizes 2,3‐oxidosqualene into lupeol and β‐amyrin at a ratio of 95 : 5. CYP716A175 catalyses the C‐28 oxidation of α‐amyrin, β‐amyrin, lupeol and germanicol, producing ursolic acid, oleanolic acid, betulinic acid, and putatively morolic acid. The gene expression of *MdOSC1* was linked to the concentrations of ursolic and oleanolic acid, whereas the expression of *MdOSC5* was correlated with the concentrations of betulinic acid and its caffeate derivatives.
- Two new multifuntional triterpene synthases as well as a multifunctional triterpene C‐28 oxidase were identified in *Malus *×* domestica*. This study also suggests that *MdOSC1* and *MdOSC5* are key genes in apple fruit triterpene biosynthesis.

## Introduction

Pentacyclic triterpenes are C30 terpenes consisting of six isoprene units. Triterpenes are distinguished by their remarkable structural diversity, with >20 000 different triterpenes reported to date (Hill & Connolly, 2013). The ursane, oleanane, and lupane series of pentacyclic triterpenes derived from α‐amyrin, β‐amyrin and lupeol, respectively, are the most widely distributed pentacyclic triterpenes in nature (Jäger *et al*., 2009). They are notably present in plant surfaces where, together with plant cuticle components, they protect the plant from water loss as well as against biotic and abiotic stresses (Gershenzon & Dudareva, 2007; Lara *et al*., 2015). A recent study on persimmon fruit (*Diospyros kaki* Thunb. cv. *Fuyu*) also revealed their importance for maintaining the mechanical strength of the cuticular matrix by functioning as nanofillers (Tsubaki *et al*., 2013). Pentacyclic triterpenes possess numerous biomedical properties (reviewed in Szakiel *et al*., 2012), including anti‐inflammatory (Andre *et al*., 2012), anti‐cancer (Salvador *et al*., 2012) and anti‐plasmodial activities (Bero *et al*., 2009). They may also serve as scaffolds for the semi‐synthesis of new lead bioactive agents. These properties are of great interest to the pharmaceutical and cosmetic industries (Moses *et al*., 2013).

**Figure 1:**
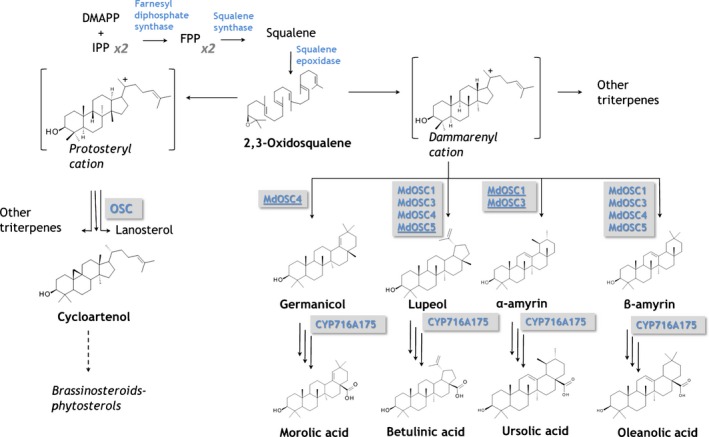
Biosynthetic pathway of triterpenic acids in apple (*Malus *× *domestica*). Triterpenes are synthesized via the mevalonic acid (MVA) pathway. The enzymes that catalyse the various steps are indicated in blue. IPP, isopentenyl diphosphate; DMAPP, dimethylallyl diphosphate; FPP, farnesyl diphosphate; OSC, oxidosqualene cyclase; CYP450, cytochrome P450. MdOSCs are underlined when they catalyse the production of the specific triterpene considered as a major component. CYP716A175 catalyses the three‐step oxidation of triterpene backbones at the C‐28 position.

The first step in the biosynthesis of all triterpenes is the cyclization of a 30‐carbon precursor, 2,3‐oxidosqualene, arising from the isoprenoid pathway (Phillips *et al*., 2006). This reaction is catalysed by oxidosqualene cyclases (OSCs or triterpene synthases), and leads to the formation of either sterol or triterpene scaffolds, in a complex series of concerted reaction steps (Fig. 1). The initial substrate folding step is critical as it will predispose the substrate to follow a particular cyclization pathway. For instance, the chair–boat–chair conformation leads to a protosteryl cation intermediate, which then gives rise to sterols, via the formation of cycloartenol in plants (Gas‐Pascual *et al*., 2014). By contrast, the chair–chair–chair conformation directs cyclization into the dammarenyl carbocation, which subsequently gives rise to diverse triterpene skeletons such as those of the ursane, oleanane, and lupane series (Xue *et al*., 2012). Approximately 80 OSCs have now been functionally characterized from plants, typically using heterologous expression of cDNAs in yeast or in tobacco (Thimmappa *et al*., 2014). OSCs are encoded by multigene families in plants. In the case of *Arabidopsis thaliana*, the genome was found to contain 13 OSC genes, encoding either monofunctional enzymes (i.e. producing a single product, e.g. cycloartenol, lanosterol, β‐amyrin, thalianol or marneral) or multifunctional enzymes producing β‐amyrin, lupeol, and a range of other products (Husselstein‐Muller *et al*., 2001; Lodeiro *et al*., 2007). Triterpene scaffolds are often further modified into more elaborate molecules by tailoring enzymes. Cytochrome P450‐mediated oxygenation of the scaffold (e.g. introduction of hydroxyl, ketone, aldehyde, carboxyl, or epoxy groups) is common and may occur at various carbon positions, for example at C‐28 for the production of triterpene acids such as ursolic acid (UA), oleanolic acid (OA) and betulinic acid (BA) (Carelli *et al*., 2011). Further steps in the triterpene synthesis pathway may also take place, including glycosylations (to form saponins) (Moses *et al*., 2014) and acylations (such as esterifications with hydroxycinnamic acids) (Andre *et al*., 2013).

In apple (*Malus* × *domestica* Borkh.), pentacyclic triterpenes account for 32–70% of the epicuticular waxes, depending on the cultivar (Belding *et al*., 1998), and their total amount may reach 60 mg per apple fruit (Andre *et al*., 2012). Ursolic and oleanolic acids, of the ursane and oleanane triterpene types, respectively, predominate in the skins of most commercial apple varieties (McGhie *et al*., 2012). A previous study showed that suberized apple skin tissue, found in partially and fully russeted old heritage varieties, has higher concentrations of lupane derivatives, including BA and specific conjugated triterpenes such as betulinic acid‐3‐*trans*‐caffeate (BA‐*trans*C) (Andre *et al*., 2013), than waxy‐skinned cultivars. In apple, three potential OSCs were identified (Brendolise *et al*., 2011) from the expressed sequence tag (EST) database constructed from cDNA libraries of ‘Royal Gala’ (Newcomb *et al*., 2006). Two of these EST sequences were essentially identical (>99% amino acid similarity; MdOSC1 and MdOSC3). MdOSC1 and MdOSC2 were produced by transient expression in *Nicotiana benthamiana* leaves and by expression in the yeast *Pichia methanolica*. MdOSC1 was shown to be a mixed amyrin synthase, with a 5 : 1 ratio of α‐amyrin to β‐amyrin. No product was evident for *MdOSC2* when it was expressed either transiently in tobacco or in yeast, suggesting that the putative triterpene synthase MdOSC2 is either encoded by a pseudogene or does not express well in these systems. This suggests that other OSC genes are present in the apple genome, to explain the concentrations of β‐amyrin and lupeol derivatives observed in apple skin (Andre *et al*., 2013). In particular, the high amount of BA found in russeted apple skin suggests the existence of a lupeol synthase, which is as yet undiscovered. Modifying enzymes responsible for the oxygenation of the triterpene scaffolds at the C‐28 position, and thereby for the occurrence of the ultimate biosynthetic products, that is, UA, OA, and BA, also need to be found.

To tackle those questions, we identified, isolated, and characterized two new multifunctional OSCs from *M. *× *domestica* (MdOSC4 and MdOSC5) using *N. benthamiana* as a heterologous system. *Nicotiana benthamiana* is naturally able to produce the triterpene precursor 2‐3 oxidosqualene, and has all the machinery required to generate triterpenes when the activity of an OSC is overexpressed (Brendolise *et al*., 2011). The activity of a cytochrome P450 (CYP716A family) responsible for the transformation of α‐amyrin, β‐amyrin, lupeol, and germanicol into their corresponding acids (Fig. 1) was confirmed using a coexpression system in *N. benthamiana*. The gene expression of four OSC genes (*MdOSC1*,* MdOSC3*, and *MdOSC4* and *MdOSC5*) as well as the cytochrome P450 was also measured in a collection of 20 contrasting apple cultivars (in terms of triterpene accumulation) and revealed the key roles played by MdOSC1 and MdOSC5 in explaining the concentrations of α‐amyrin and lupeol derivatives, respectively.

## Materials and Methods

### Chemicals

Solvents of analytical or high‐performance liquid chromatography (HPLC) grade were obtained from Thermo Fisher Scientific (Auckland, New Zealand). UA, OA and 3β‐taraxerol were purchased from Sigma‐Aldrich (St Louis, MO, USA). Amyrins, lupeol, and BA were obtained from ExtraSynthese (Genay, France). BA‐*trans*C was chemically synthesized as described in Andre *et al*. (2013).

### Plant material

For the functional characterization, apple (*Malus *×* domestica* Borkh.) cultivars ‘Merton Russet’ and ‘Royal Gala’ were used. They were grown in Hawke's Bay (New Zealand) and fruit was harvested in April 2010. Each fruit was then cut into quarters and four segment‐shaped samples (*c*. 0.5 cm at the skin edge) were cut off, avoiding sampling the core and seeds. Each segment was peeled, and the skin and the flesh were separately frozen in liquid nitrogen. Samples were stored at −80°C until analysis.

To evaluate the transcription patterns of the genes encoding different OSCs and the triterpene hydroxylase, a panel of 20 cultivars presenting various skin phenotypes, that is, from fully waxy to fully russeted exocarps, were selected. They were harvested in October 2012 in the orchards of the Walloon Agronomic Research Centre (CRA‐W, Gembloux, Belgium) at commercial maturity according to the starch iodine index (Smith *et al*., 1979). Fruit was then treated as described above for ‘Merton Russet’ and ‘Royal Gala’ and further freeze‐dried for metabolite analysis. For each cultivar and replicate (*n *=* *3), tissues from six fruits were pooled.

### Isolation and cloning of cDNAs for apple triterpene synthase and a P450

Candidate genes were first identified by a search for best similarity (blast) with known oxidosqualene cyclases from GenBank, using the Plant & Food Research EST database (Newcomb *et al*., 2006) and the apple genome (Velasco *et al*., 2010). Full‐length coding sequences for *MdOSC4* and *MdOSC5* were obtained by PCR amplification from a cDNA library made from RNA extracted from the fruit skin of ‘Merton Russet’ and ‘Royal Gala’, respectively. The resulting products were cloned into the plant transformation vector pHEX2 using Gateway reactions (Invitrogen, Mulgrave, Victoria, Australia) and transformed into *Agrobacterium tumefaciens* strain GV3101 (Hellens *et al*., 2005).

CYP716A from apple was identified by a blast search in the Plant & Food Research EST database and the genome, using the sequence of the characterized triterpene hydroxylase from *Medicago truncatula* (CYP716A12) (Fukushima *et al*., 2011). This EST was then full‐length sequenced and cloned into pSAK277S and transformed into *A. tumefaciens* strain GV3101 as described previously (Hellens *et al*., 2005).

GenBank accession numbers are as follows: *CYP716A175*, EB148173; *MdOSC1*, FJ032006; *MdOSC3*, FJ032008; *MdOSC4*, KT383435; *MdOSC5*, KT383436.

### Transient expression in *Nicotiana benthamiana*

Freshly grown transformed *A. tumefaciens* cells were re‐suspended in 10 ml of infiltration medium (10 mM MgCl_2_ and 10 μM acetosyringone) to an OD_600 nm_ of 2 and mixed 1 : 1 with *A. tumefaciens* GV3101 carrying the viral suppressor p19 (pBIN61 P19) (Hellens *et al*., 2005). Plants were grown until they had six leaves and the two to three youngest leaves over 1 cm long were infiltrated using a 1‐ml syringe and maintained in the glasshouse for the duration of the experiment. After 7 d, the leaves were detached and analysed for triterpenes. Four plants were analysed for each construct (*n *=* *4).

### Real‐time qPCR expression analysis on apple fruits

RNA was isolated using an adapted CTAB buffer extraction protocol (Gasic *et al*., 2004) as described in Legay *et al*. (2015). RNAs were cleaned and treated with DNase I using the RNeasy plant mini kit (Qiagen, Leusden, the Netherlands), following the manufacturer's recommendations. RNA integrity was assessed using the RNA Nano 6000 assay (Agilent Technologies, Diegem, Belgium) and a 2100 Bioanalyzer with quality parameters adapted to plant RNA profiles (Agilent Technologies, Santa Clara, CA, USA). Samples with RNA integrity number <7 were excluded and re‐extracted. RNA purity and concentration were assessed by measuring the absorbance at 230, 260 and 280 nm using a Nanodrop ND1000 spectrophotometer (Thermo Scientific, Villebon‐sur‐Yvette, France). cDNA synthesis was carried out on 1.5 μg of each RNA sample using M‐MuLV Reverse Transcriptase (RNase H‐), Murine RNase Inhibitor (New England Biolabs, Ipswich, MA, USA) and random hexamers (Invitrogen, Carlsbad, NM, USA) following the manufacturer's recommendations. Quantitative (q)PCR primers were designed using the primer3 software (http://frodo.wi.mit.edu/). Matching primer sets were checked using netprimer (http://www.premierbiosoft.com/net-primer/index.html) for unexpected secondary structures. In order to test the specificity of the primers, a blast search against the *M. *× *domestica* genome sequence was carried out. Primer information is available in Supporting Information Table S1. For qPCR DNA amplification, samples were run in triplicate on a Viia7 384‐well real‐time PCR instrument (Life Technologies, Carlsbad, CA, USA) using the Mesa Green Low ROX Real Time PCR kit (Eurogentec, Liege, Belgium) following the manufacturer's recommendations. Experiments were carried out following the ‘Minimum Information for Publication of Quantitative Real‐Time PCR Experiments’ (MIQE) guidelines (Bustin *et al*., 2009) and a melting curve was constructed at the end of all the runs to assess the specificity of the primers. The relative expression of a gene of interest was calculated using the biogazelle qbase+ v.2.5 software (Hellemans *et al*., 2007), taking into account multiple reference gene normalization and specific PCR efficiencies. Gene expression levels are expressed as normalized relative quantities of transcript (NRQ), using *Malus × domestica Elongation Factor 1* (*MdEF1*), *MdActin, Glyceraldehyde 3‐Phosphate DeHydrogenase* (*MdGAPDH *), and *Importin subunit alpha‐9* (*IMPA‐9*), as housekeeping genes (Vandesompele *et al*., 2002).

### Analysis of triterpenes from tobacco leaves

Powdered frozen leaf material (100 mg) was mixed with 1 ml of ethyl acetate : hexane (50 : 50 v/v). This mixture was then homogenized using a vortex for 30 s and shaken for 1 h at room temperature. After centrifugation at 10 000 ***g*** for 15 min, the supernatant was collected and evaporated to dryness using a centrifugal vacuum evaporator. The pellet was re‐extracted in 1 ml of ethanol: H_2_O (80 : 20 v/v), homogenized and shaken for 2 h at room temperature and centrifuged as described above. This supernatant was combined with the lipophilic dried supernatant and again evaporated to dryness.

Dried *N. benthamiana* extracts were made up in 500 μl of ethanol. Liquid chromatography with atmospheric pressure chemical ionisation mass spectrometry (LC‐APCI‐MS) analysis was performed using an Linear Trap Quadrupole (LTQ) ion trap mass spectrometer fitted with an atmospheric pressure chemical ionization interface (ThermoQuest, Finnigan, San Jose, CA, USA) and coupled to an Ultimate 3000 UHPLC (Dionex, Sunnyvale, CA, USA) instrument.

Compound separation for lupeol, germanicol, β‐amyrin, taraxerol and α‐amyrin was achieved isocratically on a Synergi 4μ Hydro‐RP 80 Å (Phenomenex, Torrance, CA, USA), 250 × 2 mm analytical column maintained at 50°C. Solvents were (A) H_2_O with 0.1% formic acid and (B) CH_3_CN and the flow rate was 300 μl min^−1^ at 85% B. The injection volume was 5 μl. Compound separation for BA, OA, and UA was achieved isocratically on a Poroshell 120 SB‐C18 2.7μ (Agilent, Santa Clara, CA, USA), 150 × 2.1 mm analytical column maintained at 70°C. Solvents were (A) H_2_O with 0.2% (v/w) ammonium acetate and (B) MeOH : H_2_O with 0.2% (v/w) ammonium acetate (83 : 17, v/v) and the flow rate was 200 μl min^−1^ at 92% B. The injection volume was 2 μl.

MS data were acquired in the positive mode using a data‐dependent LC‐MS^3^ method. Lupeol, β‐amyrin, α‐amyrin, taraxerol, UA, OA and BA were identified by their retention times and spectral data compared with authentic standards and were quantified by monitoring the *m/z* 409.8 [MH‐H_2_O]^+^ ion for lupeol, β‐amyrin, α‐amyrin, and taraxerol and the sum of three ions for UA, OA and BA, *m/z* 474.6 [M+NH_4_]^+^, *m/z* 456.6 [M]^+^ and *m/z* 439.8 [MH‐H_2_O]^+^. External quantification was used. Putative compounds germanicol and morolic acid were quantified as equivalents of β‐amyrin and OA, respectively. Ratios of the different metabolites produced from OSC transient expression were calculated as a percentage of the total identified triterpenes.

### Analysis of triterpenes from apple fruits

Powdered freeze‐dried material (500 mg) was mixed with 10 ml of ethyl acetate : hexane (50 : 50 v/v). This mixture was then homogenized using a vortex for 30 s and shaken for 1 h at room temperature. After centrifugation at 10 000 ***g*** for 15 min, the supernatant was collected and evaporated to dryness using a centrifugal vacuum evaporator. The pellet was re‐extracted using 10 ml of ethanol : H_2_O (80 : 20 v/v), homogenized and shaken for 2 h at room temperature and centrifuged as described above, or 10 000 ***g*** for 15 min. The supernatant was collected, combined with the lipophilic dried extract and evaporated to dryness. Triterpenes were re‐suspended in 1 ml of EtOH and filtered (0.45 μm) before HPLC analysis. Each cultivar of apple was analysed in triplicate (*n *=* *3).

High‐performance liquid chromatography with diode‐array detection (HPLC‐DAD) analysis of triterpenes was carried out on a Thermo Separation Product system (Thermo Scientific, San Jose, CA, USA) equipped with a P200 pump, an AS100 autosampler, an SN4000 interface, and a UV6000LP DAD, using a C18 (2) Luna (250 × 4.6 mm ID; 5 μm particle size) (Phenomenex, Torrance, CA, USA) column. The mobile phase was MeOH : H_2_O : phosphoric acid (88 : 11.95 : 0.05, v/v/v). The injection volume was 20 μl. Triterpenes were eluted isocratically at a flow rate of 1 ml min^−1^ and a column temperature of 35°C. Identification and quantification of triterpenes were performed as described previously (Andre *et al*., 2013). UA, OA, BA and BA‐*trans*C were identified by their retention time and spectral data compared with authentic standards and were quantified at 210 nm (UA, OA, and BA) and 320 nm (BA‐*trans*C) using five‐point calibration curves. Excellent linearity (*R* ^2^ > 0.99) was obtained in the concentration range 100–6.25 μg ml^−1^ for all compounds. Betulinic acid‐3‐*cis*‐caffeate and oleanolic acid‐3‐*trans*‐caffeate were quantified at 320 nm as BA‐*trans*C equivalent.

### Phylogenetic analysis

The alignment and phylogenetic analysis of the OSC and cytochrome P450 protein sequences were performed with the geneious pro 4.8.5 software (Biomatters Ltd, Auckland, New Zealand). The consensus method used was moderate greedy clustering. The neighbour‐joining consensus tree was built on the protein alignment.

### Statistical analysis

Principal component analysis (PCA) was carried out to determine the relationships among the metabolite data in the collection of 20 cultivars. The software past 3.x was used for the PCA (Hammer *et al*., 2001), while sigmaplot v.12.5 (Systat Software Inc., San Jose, CA, USA) was used for mean comparisons of triterpene contents.

## Results and Discussion

### Cloning and sequence analysis of OSCs from apple

We previously identified contrasting triterpene profiles in the apple skins of the cultivars ‘Royal Gala’ and ‘Merton Russet’ (Andre *et al*., 2013). In particular, the waxy skin of ‘Royal Gala’ showed high amounts of UA and OA, whereas the russeted skin of ‘Merton Russet’ was characterized by higher concentrations of lupeol derivatives, including BA. This differential accumulation gave us a new opportunity to better understand triterpene regulation in apple and, in particular, to discover a previously undescribed lupeol synthase. The synthesis of UA and OA precursors, α‐ and β‐amyrin, respectively, could indeed be partly attributed to the expression of the previously described *MdOSC1* and *MdOSC3* genes (Brendolise *et al*., 2011). The transient expression of *MdOSC3* in *N. benthamiana* confirmed the functional similarity of MdOSC1 and MdOSC3 postulated by (Brendolise *et al*., 2011). α‐Amyrin and β‐amyrin were indeed produced at high concentrations, together with a small amount of lupeol, at a ratio of 85 : 14 : 1 (Fig. S1).

**Figure 2:**
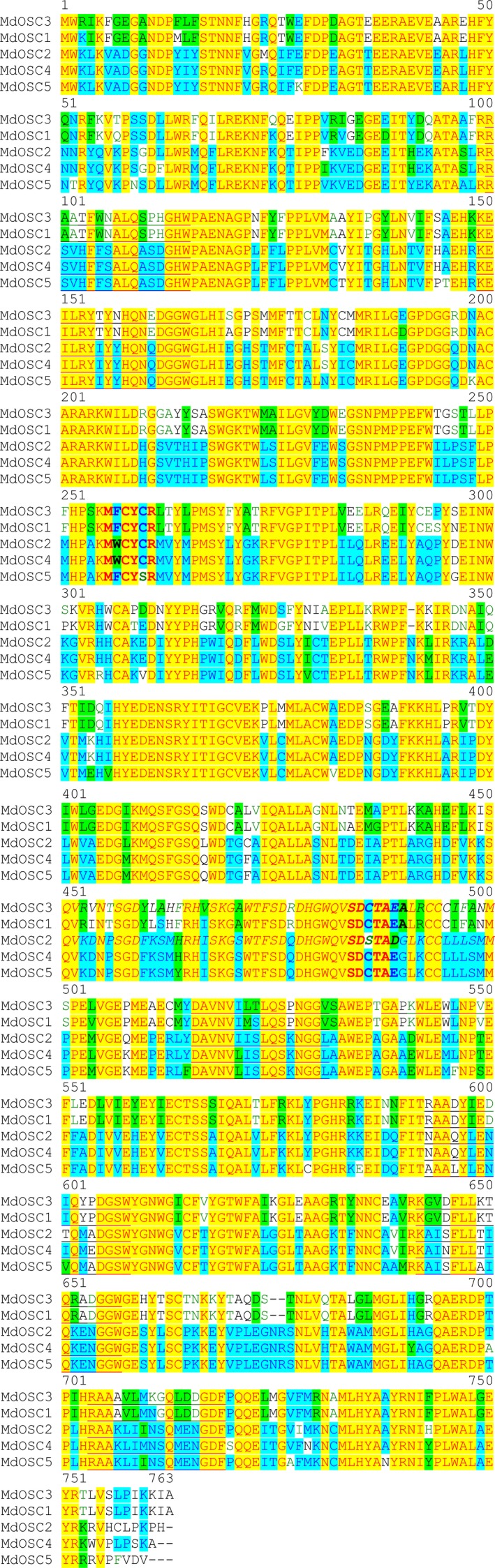
Alignment of oxidosqualene cyclase (OSC) predicted amino acid sequences from apple (*Malus *× *domestica*). The conserved DCTAE as well as the M(W/Y)CY(C/S)R sequences are shown in bold (residues 485 onwards and 256 onwards, respectively). Six of the QW motifs (Q‐X3‐G‐X‐W) (Siedenburg & Jendrossek, 2011) are underlined. The text/background colour code refers to the degree of similarity between the different sequences: red/yellow, identical; dark blue/turquoise, conservative; black/green, a block of similar sequences; black/white, nonsimilar.

**Figure 3:**
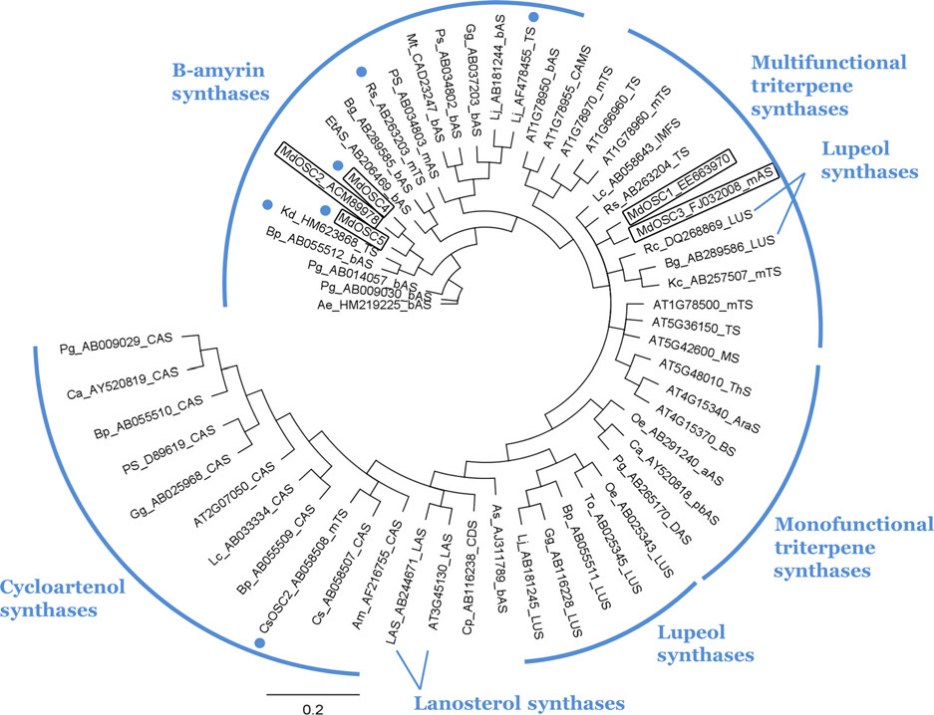
Phylogenetic tree of a wide range of triterpene synthase amino acid sequences, built using the neighbour‐joining method. Sequences were selected from GenBank based on their authentication in the literature (unless otherwise indicated). The scale bar indicates 0.2 amino acid substitutions per site. The GenBank or The Arabidopsis Information Resource (TAIR) identifier is included in the gene name. The two‐letter prefix for each name in the tree identifies the species name as follows: Ab, *Abies magnifica*; Ae, *Aralia elata*; As, *Avena strigosa*; Bg, *Bruguiera gymnorhiza*; Bp, *Betula platyphylla*; Ca, *Centella asiatica*; Cp, *Cucurbita pepo*; Cs, *Costus speciosus*; Et, *Euphorbia tirucalli*; Gg, *Glycyrrhiza glabra*; Kc, *Kandelia candel*; Kd, *Kalanchoe daigremontiana*; Lc, *Luffa cylindrical*; Lj, *Lotus japonicas*; Mt, *Medicago truncatula*; Oe, *Olea europaea*; Pg, *Panax ginseng*; Rc, *Ricinus communis*; Rs, *Rhizophora stylosa*; To, *Taraxacum officinale*. Measured triterpene activities are encoded in the suffix for each entry name as follows: aAS, alpha amyrin synthase; AraS, arabidiol synthase; bAS, beta‐amyrin synthase; BS, baruol synthase; CAMS, camelliol c synthase; CAS, cycloartenol synthase; CDS, cucurbitadienol synthase; DAS, dammarenediol‐II synthase; IMFS, isomultiflorenol synthase; LAS, lanosterol synthase; LUS, lupeol synthase; mAS, mixed amyrin synthase (both alpha and beta amyrin); MS, marneral synthase; mTS or TS, multifunctional terpene synthase; pbAS, putative bAS; ThS, thalianol synthase. Multifunctional triterpene synthases within monofunctional triterpene synthase groups are marked with a bullet.

An OSC cyclizing 2,3‐oxidosqualene mostly to lupeol has never been described in apple. Candidate genes were first identified by a search for best similarity (blast) with lupeol synthases from GenBank, using the Plant & Food Research EST database (Newcomb *et al*., 2006) and the apple genome (Velasco *et al*., 2010). Two candidates (*MdOSC4* and *MdOSC5*) were identified and the level of their gene expression was confirmed by qPCR to be higher in ‘Merton Russet’ than in ‘Royal Gala’ (nine‐fold and two‐fold, respectively; *P *<* *0.01; Fig. S2). Candidates were further isolated and amplified by PCR from a cDNA library made from RNA extracted from the fruit skin of ‘Merton Russet’ (*MdOSC4*) and ‘Royal Gala’ (*MdOSC5*). The predicted open reading frames (ORFs) of *MdOSC4* and *MdOSC5* encode proteins of 761 and 760 amino acids, respectively (Fig. 2). These two protein sequences are 93% identical at the amino acid level and 94% identical at the DNA level within the coding sequences. MdOSC4 and MdOSC5 are only 64–65% identical to MdOSC1 at the protein level and 70–71% identical at the DNA level. Like other OSCs, MdOSC4 and MdOSC5 contain the highly conserved DCTAE motif, which is implicated in substrate binding (Fig. 2), and six repeats of the QW motifs, typical of the triterpene synthase superfamily (Siedenburg & Jendrossek, 2011). It has been suggested that the QW motifs may strengthen the structure of the enzyme and stabilize the carbocation intermediates during cyclization (Kushiro *et al*., 2000). Analysis of the apple genome revealed that *MdOSC4* and *MdOSC5* are located in linkage groups LG9 and LG17, respectively. These chromosomes are homologous, resulting from whole‐genome duplication (Velasco *et al*., 2010). Examination of the apple genome shows that the apple OSC genes have at least 14 exons, while typical *A. thaliana* triterpene cyclases have 17 exons (Husselstein‐Muller *et al*., 2001). Phylogenetic analysis showed that MdOSC4 and MdOSC5 lie among the β‐amyrin synthases and other mixed functional triterpene synthases and away from identified lupeol synthases (Fig. 3).

The results of this search for lupeol synthases as well the availability of the apple genome led us to a reappraisal of MdOSC2 as a valid OSC. Transient expression of the *MdOSC2* gene in yeast and *N. benthamiana* revealed that *MdOSC2* was inactive. Interestingly, MdOSC2 and MdOSC4 are 96% identical at the amino acid level. They both match the same region of the genome on chromosome 9 (LG9) (sharing 94% and 96% homology with MDP0000034165, respectively, at the amino acid level). Therefore, none of these three sequences are identical, which could be attributable to genotypic variability, as *MdOSC2* was isolated from ‘Royal Gala’, *MdOSC4* was isolated from ‘Merton Russet’, and the genome was sequenced from ‘Golden Delicious’, or to allelic variation within the same cultivar at the same locus. It is unlikely that the differences are attributable to cloning issues, as high‐fidelity polymerase was used.

When the amino acid sequences of MdOSC2 and MdOSC4 were carefully compared, differences appeared at key positions in the protein. First of all, the motif SDCTAE, which is essential for substrate binding (Poralla, 1994) is not conserved in MdOSC2 and is replaced by SDSTAD. Although the substituted amino acids share similar properties (Betts & Russell, 2003), we cannot exclude the possibility that these modifications have an influence on the inactivity of MdOSC2. Then, a potentially important difference was found in one of the QW motifs, which are imperative for stabilizing the carbocation intermediates during cyclization (Kushiro *et al*., 2000). MdOSC4, like numerous OSCs, possesses an isoleucine at position 601, a hydrophobic amino acid, whereas MdOSC2 has a threonine (small polar amino acid). Further site‐directed mutagenesis experiments would, however, be needed to draw conclusions about the influence of these amino acid changes on the functionality of MdOSC2 and MdOSC4.

### Functional characterization of MdOSC4 and MdOSC5 in *Nicotania benthamiana*

**Figure 4:**
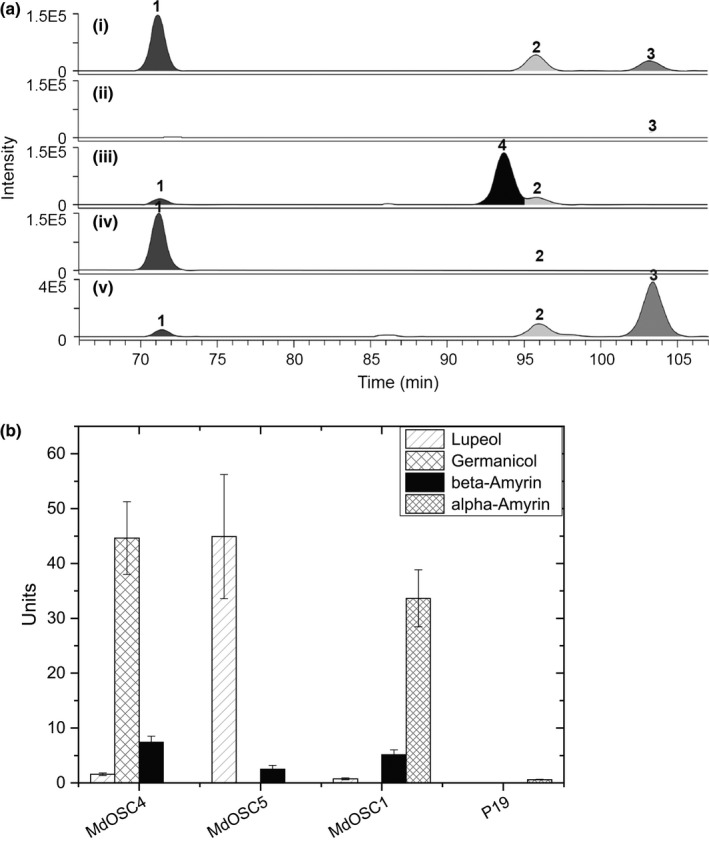
Triterpene synthesis in tobacco leaves by heterologous expression of candidate triterpene synthases. (a) Chromatograms of typical LC‐MS analysis of the products of (iii) *Malus *× *domestica OXIDOSQUALENE CYCLASE4* (*MdOSC4*), (iv) *MdOSC5*, and (v) *MdOSC1* after transient expression in *Nicotiana benthamiana*. p19 was used as a negative control (ii) and mixed with authentic standards at 20 μg ml^−1^ (1, lupeol; 2, β‐amyrin; 3, α‐amyrin) (i). Peak 4 on trace (iii) is the putative identification of germanicol. Compounds were identified and quantified on the basis of their mass spectral data (Supporting Information Figs S3, S4). Chromatograms are presented as selected ion plots of the *m*/*z* 409.8 [MH‐H_2_O]^+^ ion. (b) Quantification of triterpene production by the various triterpene synthase genes in tobacco. Data show nmoles of triterpenes produced from 100 mg of extracted tissue (mean with SE; *n *=* *4). Germanicol was quantified as β‐amyrin equivalents.

To identify the products of the putative OSCs, heterologous expression was carried out in tobacco (*N. benthamiana*). The full‐length cDNAs of the two putative enzymes were cloned into a plant transformation vector and transformed into *A. tumefaciens*. *Agrobacterium tumefaciens* cells were injected into tobacco leaves, which were harvested after 7 d and processed for extraction of triterpenes. The production of triterpenes in tobacco leaves for the two new triterpene synthases (MdOSC4 and MDOSC5) as well as for the previously described MdOSC1 is shown in Fig. 4. The triterpene products were identified by LC‐MS by comparing their retention times and MS characteristics with those of authentic standards for α‐amyrin, β‐amyrin and lupeol (Figs 4a, chromatogram I, S3).

Three unique peaks were observed in the chromatogram of tobacco leaves in which *MdOSC4* was transiently expressed (Fig. 4aiii), whereas they were not detected in the p19 control (Fig. 4aii). The predominant component was identified as germanicol, an olean‐18‐ene triterpene, by comparison of its retention time and MS spectra with data from the literature (Budzikiewicz *et al*., 1963; Kawano *et al*., 2002) (Fig. S3). We could exclude the assignment of this molecule to taraxerol, a closely related compound, by comparison with an available standard molecule (Fig. S4). The expression of *MdOSC4* led also to the formation of smaller peaks corresponding to β‐amyrin and lupeol. As a result, the accumulation of germanicol : β‐amyrin : lupeol was in the proportion 82 : 14 : 4 (Fig. 4b). This is the first report of a germanicol synthase in *M. *× *domestica*. OSCs synthesizing germanicol as the predominant component have rarely been described in the plant kingdom. Only the multifunctional triterpene synthase from *Rhizophora stylosa* (RsM1), sharing 80% homology with *MdOSC4* at the DNA level, has been shown in yeast to transiently produce germanicol, β‐amyrin and lupeol in similar proportions (63 : 33 : 4) (Basyuni *et al*., 2007). Other known OSCs produce germanicol to a lesser extent, including *CsOSC2* from *Costus speciosus*, predominantly catalysing the formation of β‐amyrin, followed by germanicol, lupeol and other minor products (Kawano *et al*., 2002), and the lupeol synthase 1 (LUP1) from *A. thaliana* (Segura *et al*., 2000).

Cells transiently expressing *MdOSC5* cDNA were found to generate lupeol as a major component and β‐amyrin as a minor product, in a ratio of 95 : 5 (Fig. 4aiv). This is the first report of an OSC catalysing the formation of lupeol in apple. Similar lupeol synthases have been described in various species (Thimmappa *et al*., 2014), including the enzyme *KdLUP* from *Kalanchoe daigremontiana* (Wang *et al*., 2010), which produces lupeol and β‐amyrin in the same proportion as *MdOSC5*.

Expression of the previously characterized *MdOSC1* (Brendolise *et al*., 2011) confirmed its multifunctionality, producing predominantly α‐amyrin and β‐amyrin to a lesser extent (Fig. 4av). In contrast with our previous study (Brendolise *et al*., 2011), small amounts of lupeol were also detected, signifying a new α‐amyrin : β‐amyrin : lupeol production ratio for *MdOSC1* of 85 : 13 : 2 (Fig. 4b).

### Cloning and sequence analysis of a P450 from apple

**Figure 5:**
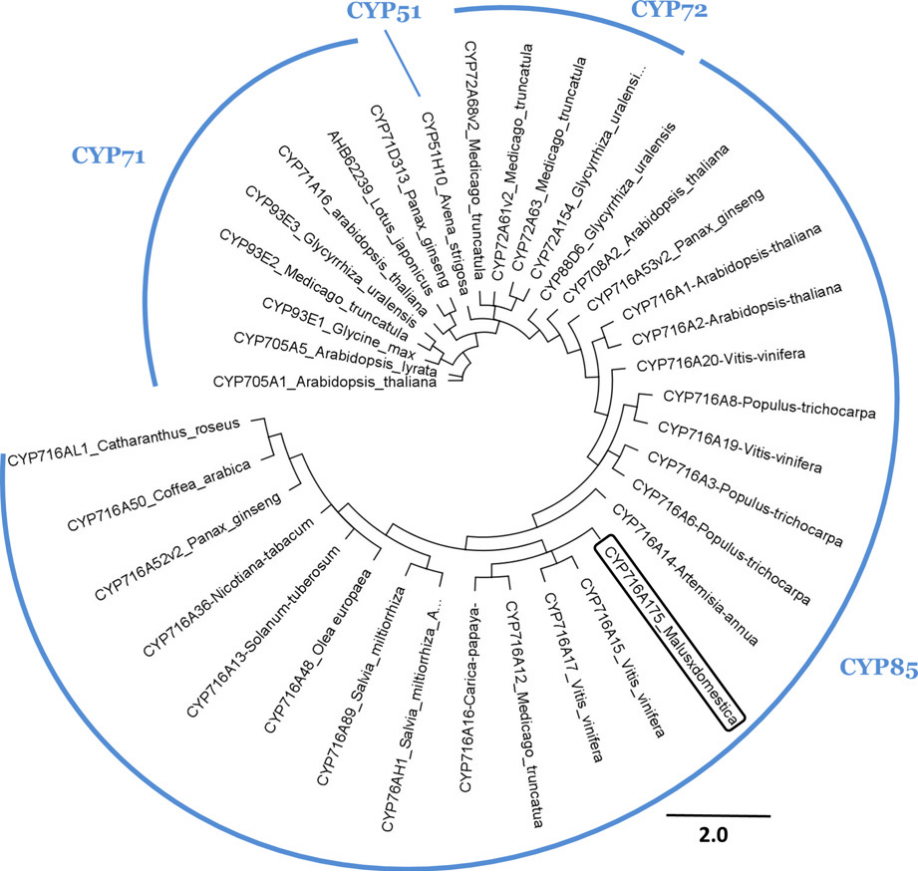
Neighbour‐joining tree of a wide range of triterpene‐related P450 amino acid sequences. Note that the apple cytochrome P450 (CYP716A175) is close to the *Medicago truncatula* authenticated triterpene C‐28 oxidase (CYP716A12). The prefix is the GenBank or database identifier. The scale bar indicates 2 amino acid substitutions per site. P450s are clustered according to the P450 clan they belong to.

In apple skin, the triterpene scaffolds α‐amyrin, lupeol and β‐amyrin, produced through cyclization of 2,3‐oxidosqualene, do not accumulate in high amounts, and are mainly found in their acidic forms, that is, UA, OA, and BA, respectively. This reaction is probably catalysed by cytochrome P450‐dependent monooxygenases (P450s) through a three‐step reaction: addition of a hydroxyl group at C‐28 that is further oxidized to an aldehyde and then to a carboxyl group (Fukushima *et al*., 2011). To date, no P450 has been identified in apple that carries out this reaction. In light of the large number of members and significant diversity within the CYP multigene family (Pateraki *et al*., 2015), we looked for homologues of *CYP716A12*, a known C‐28 oxidase isolated from *M. truncatula*, that converted α‐amyrin to UA, β‐amyrin to OA and lupeol to BA (Fukushima *et al*., 2011). This sequence was used to identify the closest sequence in apple, which was annotated *CYP716A175* by the P450 nomenclature committee (Fig. 5). The DNA sequence identity of the coding sequence of *CYP716A175* (GenBank accession EB148173) with the *M. trunculata CYP716A12* was 72% and was the best blast match available in the Plant & Food Research EST libraries (Newcomb *et al*., 2006). *CYP716A175* encodes a protein of 484 amino acids and is located in linkage group 13 (LG13) in the genome. An alignment of *CYP716A175* with putative C‐28 triterpene hydroxylases from other species is shown in Fig. S5 and shows that *CYP716A175* is clearly grouped with the *CYP716A* subfamily (Fig. 5).

### Functional characterization of the P450

**Figure 6:**
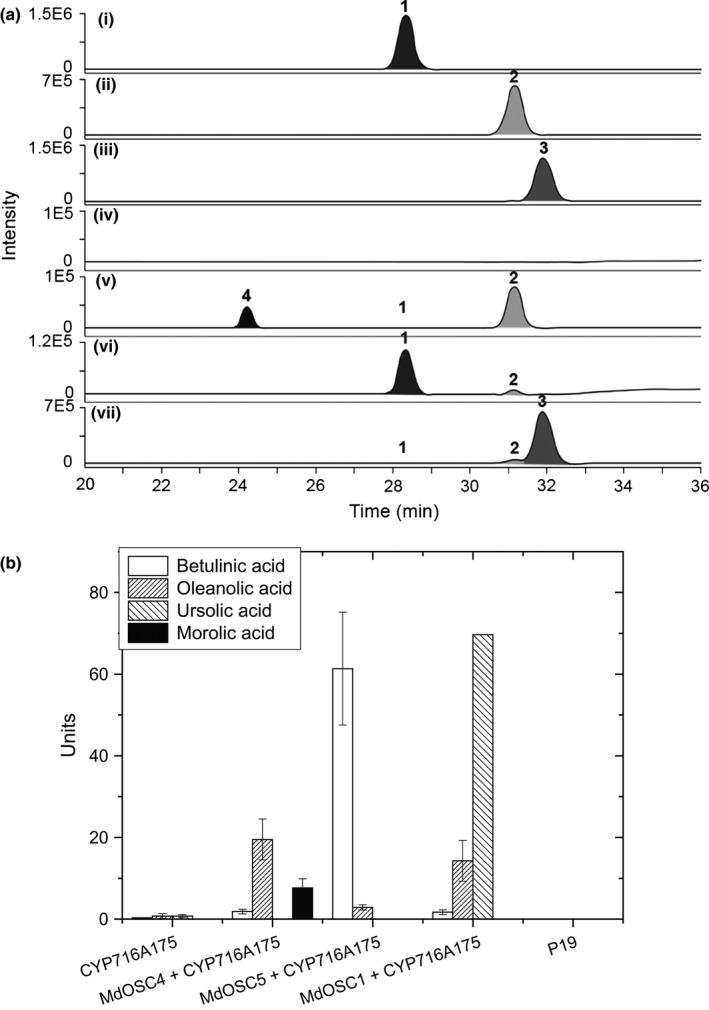
Triterpene acid synthesis in tobacco leaves by heterologous coexpression of a cytochrome P450 (CYP) gene with triterpene synthases. (a) Chromatograms of typical LC‐MS traces of the products of *CYP716A175* (P450) coexpressed with (v) *Malus *× *domestica OXIDOSQUALENE CYCLASE4* (*MdOSC4*), (vi) *MdOSC5*, and (vii) *MdOSC1*. p19 was used as a negative control (iv) and compared with authentic standards of (i) betulinic acid (1) at 100 μg ml^−1^; (ii) oleanolic acid (2) at 100 μg ml^−1^, and (iii) ursolic acid (3) at 100 μg ml^−1^. (4) Putative identification of morolic acid. Compounds were identified and quantified on the basis of their mass spectral data (Supporting Information Fig. S6). Chromatograms are presented as selected ion plots of the sum of three ions: *m*/*z* 474.6 [M+NH_4_]^+^, *m*/*z* 456.6 [M]^+^ and *m*/*z* 439.8 [MH‐H_2_O]^+^. (b) Quantification of the amount of triterpene acids made by the addition of a P450 gene to the triterpene synthases. Morolic acid was quantified as oleanolic acid equivalent. Data are nmoles of triterpenes produced from 100 mg of extracted tissue (mean ± SE; *n *=* *4).

To confirm the functional activity of our P450 candidate, we transiently expressed it in tobacco leaves together with our previously identified OSCs. The LC‐MS analysis of the organic extract of the 7‐d‐old leaves revealed that this enzyme could transform the four triterpene scaffolds, that is, α‐amyrin, β‐amyrin, lupeol and germanicol, into their corresponding C‐28 acids (Fig. 6). The presence of UA, OA and BA could thus be confirmed by comparison with the retention times and spectral data of authentic standards (Fig. S6). It appeared also that this multifunctional P450 is able to convert germanicol into morolic acid, which corresponds to germanicol with a carboxyl group at position 28 (peak 4, Fig. 6a, chromatogram vi). The spectral data for the putative morolic acid are presented in Fig. S6, but could not be compared with a standard. It is noteworthy that no triterpenes remained when both the triterpene synthase and the P450 were added together, suggesting that the P450 converted all the triterpenes to their respective C‐28 acids. The CYP716A175 identified here as a C‐28 oxidase has similar activities to its close homologues from other plant species: *Panax ginseng* (Han *et al*., 2013), *Vitis vinifera* (Fukushima *et al*., 2011), *M. truncatula* (Carelli *et al*., 2011), *Maesa lanceolata* (Moses *et al*., 2015) and *Catharanthus roseus* (Huang *et al*., 2012).

Transient expression of *MdOSC1* and *MdOSC5* together with *CYP716A175* confirmed the ratio between the different triterpene backbones observed after transient expression of *MdOSC1* and *MDOSC5* alone. However, the ratio between the acid versions of germanicol, β‐amyrin, and lupeol was not conserved after transient expression of *MdOSC4* with the P450. The ratio of accumulation of morolic acid : OA : BA was 31 : 63 : 6, whereas the ratio of their precursors germanicol : β‐amyrin : lupeol was 82 : 14 : 4. Several hypotheses could explain this result: the ionization efficiency of morolic acid under our MS conditions is different from that of OA; CYP716A175 has a similar oxidizing capacity for the triterpene scaffold α‐amyrin, β‐amyrin, and lupeol but not for germanicol; some native tobacco genes could induce other types of modification in morolic acid and produce metabolites that were not detected in our analytical method; or the apple CYP716A175 initiates a catalytic cascade by introducing the first hydroxyl at C‐28, which is then oxidized by the native tobacco machinery. The first hypothesis could be tested by the use of authentic standards for germanicol and morolic acid to establish their relative response factors, as these compounds were quantified in the current investigation as β‐amyrin and OA, respectively. The second theory could be practically tested by expressing *CYP716A175* with several monofunctional OSCs that make specific products and determining its catalytic efficiency for each substrate. It is also conceivable that the higher oxidizing efficiency of CYP716A175 for lupeol and β‐amyrin compared with germanicol preferentially drives the whole reaction chain to the production of BA and OA rather than morolic acid. The third hypothesis could be tested in a metabolomics study on various extracts. Whereas the last assumption could be valid for germanicol, as the current study describes its conversion to morolic acid for the first time, this is unlikely to occur for the other triterpene backbones. First, our data match data from the literature concerning the three‐step oxidation reaction catalysed by a CYP1716A enzyme, leading to the conversion of lupeol, α‐amyrin and β‐amyrin into their corresponding C‐28 acids: BA, UA, and OA (Carelli *et al*., 2011; Fukushima *et al*., 2011). In addition, CYP716A175 clusters together with other P450 C‐28 oxidases, as shown in the phylogenetic analysis (Fig. 5). Functional characterization of related CYP716As has been performed using various heterologous expression systems, and it appears that the product profile is influenced by both the C‐28 carboxylation capacity of the enzyme and the heterologous host. Heterologous expression in yeast of *CYP716A12* from *M. truncatula* and *CYP716A75* from *Maesa lanceolata* with a β‐amyrin synthase (Moses *et al*., 2015) has revealed the accumulation of oleanolic OA, but also of its intermediates erythrodiol and oleanolic aldehyde, in the ratios 1.1 : 6.5 : 2.4 and 0.2 : 8.7 : 1.1, respectively. This result confirmed the sequential three‐step oxidation reaction leading to OA as well as the low efficiency of these enzymes (and especially of CYP716A75) to oxidize erythrodiol in yeast. A recent study functionally characterized two CYP716As (CYP716A80 and CYP716A81) from *Barbarea vulgaris* in both yeast and *N. benthamiana* (Khakimov *et al*., 2015). Transient expression in *Saccharomyces cerevisiae* showed the presence of the intermediate erythrodiol in addition to OA, but not of oleanolic aldehyde. In agreement with our study, no intermediates were identified after 7 d of transient expression in tobacco of a β‐amyrin synthase with *CYP716A80* and *CYP716A81*; only OA, along with some minor unknown metabolites, was detected (Khakimov *et al*., 2015). However, unknown metabolites were also produced to an extent depending on the *CYP716A* considered. *In vitro* experiments performed with *CYP716A*‐expressing microsomes produced OA only (Carelli *et al*., 2011; Fukushima *et al*., 2011; Han *et al*., 2013; Khakimov *et al*., 2015).

### Expression patterns of triterpene‐related genes in different apple cultivars

**Figure 7:**
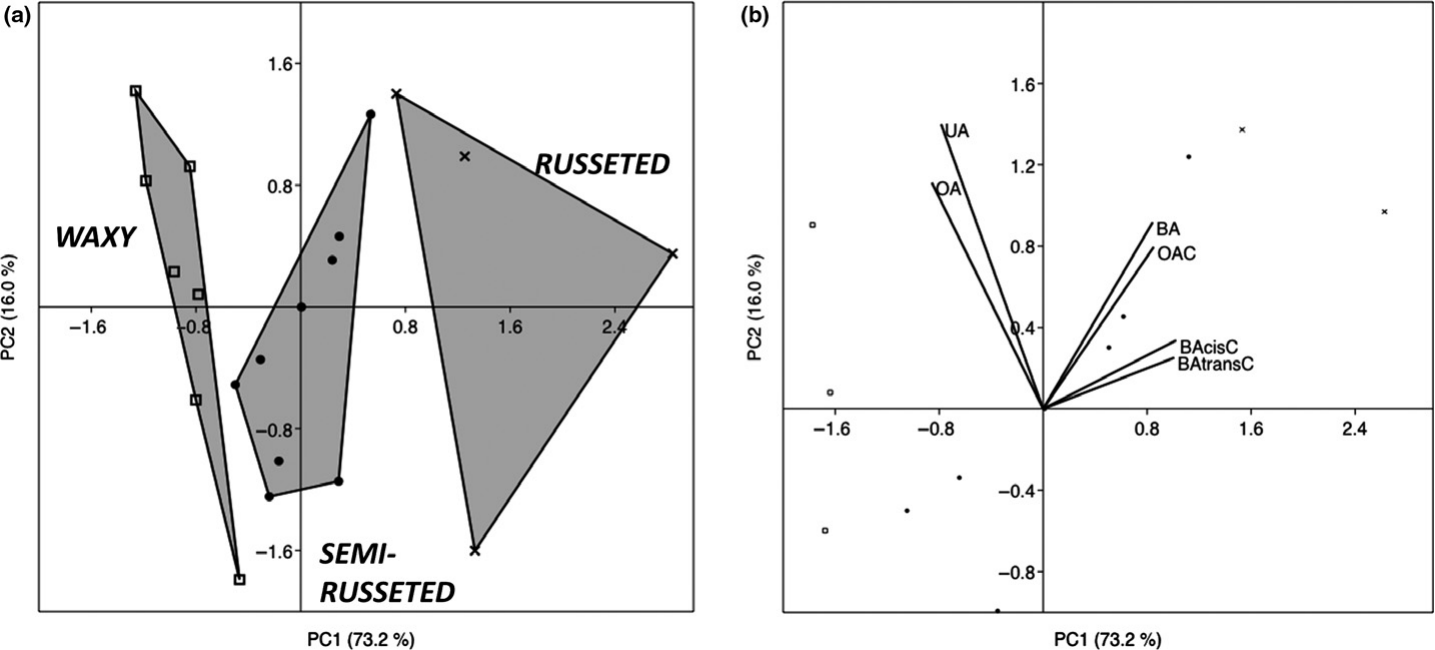
Principal component analysis (PCA) performed on 20 individuals (apple cultivars) and six triterpene concentrations. (a) Score plot of the 20 cultivars separating into three russeting groups: russeted, semi‐russeted, and waxy. (b) Loading plot showing the relationships among phytochemical data. UA, ursolic acid; BA, betulinic acid; BA‐cisC, betulinic acid‐3‐*cis*‐caffeate; BA‐transC, betulinic acide‐3‐*trans*‐caffeate.

In order to evaluate the contributions of the identified OSCs to the accumulation of triterpenes in apple skins, metabolite and transcriptomic data were collected from a panel of 20 apple cultivars presenting contrasting skin phenotypes: waxy, semi‐russeted or russeted skin. Six major triterpenes were identified and quantified using HPLC‐DAD (Tables 1, S2): three triterpene acids (UA, OA and BA) and three triterpene caffeates (BA‐*trans*C, oleanolic acid‐3‐*trans*‐caffeate and betulinic acid‐3‐*cis*‐caffeate). Morolic acid was not detected in these apple samples. There was no significant difference between the three apple phenotypic groups in terms of total triterpenes (Tables 1, S2). Analysis of individual triterpene components revealed, however, large profile variabilities: UA and OA were significantly predominant in waxy apple skins compared with russeted ones (2839 vs 1569 and 2045 vs 800 nmol g^−1^ DW, respectively), whereas BA significantly dominated in russeted apples compared with waxy ones (1628 vs 687 nmol g^−1^ DW, respectively); the concentrations of triterpene‐caffeates were higher in russeted apples than in waxy apples (2902 vs 294 nmol g^−1^ DW, respectively). A principal component analysis (PCA) was performed on the 20 apple cultivars (from three phenotypic groups) and six variables consisting of the concentrations of six triterpenes (Fig. 7a,b). Principal component 1 (PC1) and PC2 explained 73.2% and 16.0% of the variability, respectively. PC1 clearly separated the three phenotypic groups (waxy, semi‐russeted, and russeted) along its axis (Fig. 7a). Two positive correlations were noted: between waxy apples and the concentrations in UA and OA; and between russeted apples and the contents in BA and triterpene‐caffeates (Fig. 7a). As expected, the semi‐russeted group of cultivars showed an intermediary position between these two contrasting groups. These results therefore confirm the association between apple russetting and specific skin triterpene composition at maturity that we observed in our previous study (Andre *et al*., 2013).

**Table 1:** Triterpene composition of 20 apple cultivars separated into three russeting groups: russeted (four cultivars), semi‐russeted (nine cultivars), and waxy (seven cultivars)

Cultivars	Triterpene acids (nmol g^−1^ DW)			Triterpene‐caffeates (nmol g^−1^ DW)		
	Ursolic acid	Oleanolic acid	Betulinic acid	Oleanolic acid‐3‐*trans*‐caffeate	Betulinic acid‐3‐*trans*‐caffeate	Betulinic acid‐3‐*cis*‐caffeate
Russeted						
St Edmund's Pippin	2059 ± 70	922 ± 160	2063 ± 578	412 ± 22.4	1402 ± 60.9	292 ± 15.6
Court Pendu Gris	743 ± 59	500 ± 74	1094 ± 61	248 ± 4.4	2048 ± 23.8	3389 ± 11.0
Reinette Parmentier	2802 ± 151	1270 ± 88	1262 ± 128	511 ± 20.6	1287 ± 54.1	266 ± 11.9
Patte de loup	670 ± 50	510 ± 59	2095 ± 124	493 ± 11.0	3722 ± 97.8	586 ± 28.3
Semi‐russeted						
Reinette de Blenheim	1819 ± 41	1240 ± 207	527 ± 70.2	169 ± 27.0	369 ± 25.3	105 ± 26.7
Wellant	2026 ± 98	1721 ± 173	1059 ± 154	185 ± 9.6	804 ± 5.3	176 ± 7.2
Reinette Professeur Lecrenier	1464 ± 112	991 ± 134	1153 ± 250	154 ± 2.3	719.6 ± 9.7	147 ± 11.3
Reinette de France	1727 ± 270	1312 ± 163	1091 ± 303	72 ± 8.1	396 ± 24.7	88.9 ± 26.4
Coxigold	2100 ± 135	1974 ± 428	1712 ± 729	282 ± 12.7	1312 ± 26.6	256 ± 13.5
CRA‐W/Ma/AA125	1962 ± 216	1848 ± 210	884 ± 440	95 ± 2.2	291 ± 11.5	68 ± 6.4
Reinette de Hollande	2503 ± 66	1319 ± 244	575 ± 117	270 ± 31.6	359 ± 7.3	82 ± 7.3
Reinette de Chenée	2263 ± 61	1523 ± 211	900 ± 158	406 ± 5.7	1041 ± 16.6	178 ± 2.9
CRA‐W/Ma/AC22	1964 ± 319	1710 ± 474	1354 ± 311	208 ± 14.4	949 ± 27.6	201 ± 9.6
Waxy						
CRA‐W/Ma/AF34	1680 ± 97	1203 ± 125	324 ± 100	114 ± 13.5	185 ± 21.9	56.6 ± 38.1
CRA‐W/Ma/AG94	3165 ± 249	2264 ± 152	744 ± 200	175 ± 12.6	252 ± 21.4	72.6 ± 21.0
Topaz	2942 ± 301	2057 ± 366	778 ± 218	58.8 ± 9.1	82.2 ± 13.0	34.5 ± 9.3
CRA‐W/Ma/AF42	3816 ± 498	2540 ± 372	734 ± 49.2	82.0 ± 4.1	161 ± 16.5	45.0 ± 14.5
Royal Gala	2756 ± 103	1917 ± 209	693 ± 246	129 ± 11.8	178 ± 26.1	48.4 ± 22.8
Lord Lambourne	3265 ± 178	2472 ± 304	834 ± 106	22.9 ± 1.2	121 ± 6.1	34.3 ± 0.2
CRA‐W/Ma/AM84	2249 ± 127	1862 ± 391	700 ± 173	47.6 ± 2.8	135 ± 11.1	25.3 ± 0.6

Data are expressed in nmol g^−1^ DW (mean ± SD; *n *=* *3). Data were obtained after HPLC‐DAD analysis as described in the Materials and Methods section.

**Figure 8:**
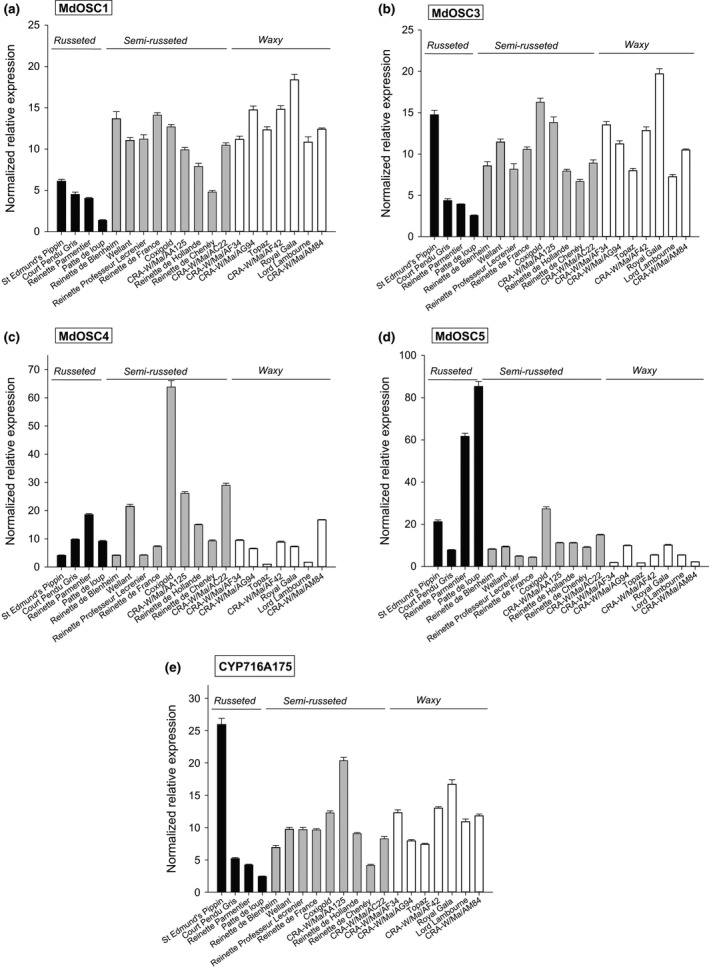
Expression analysis of triterpene biosynthetic genes (a, *Malus *× *domestica OXIDOSQUALENE CYCLASE1* (*MdOSC1*); b, *MdOSC3*; c, *MdOSC4*; d, *MdOSC5*; e, *CYP716A175*) by real‐time quantitative (q)PCR on RNA extracted from apple skin tissues. The normalized relative expression was rescaled to the sample with the lowest relative expression, that is, the expression level was set to 1 in the cultivar ‘Topaz’ for *MdOSC4*. Error bars show SD (*n *=* *3).

The transcription levels of the five genes under investigation were then assessed by RT‐qPCR: four oxydosqualene cyclases (*MdOSC1*,* MdOSC3*,* MdOSC4*, and *MdOSC5*), and the triterpene monooxygenase (*CYP716A175*). Gene expression levels differed greatly according to the apple cultivar and the gene measured (Fig. 8). The largest variation among genotypes was observed for the *MdOSC4* and *MdOSC5* genes, with a 63‐fold lower expression in the waxy skinned ‘Topaz’ than in the semi‐russeted ‘Coxigold’ and 50‐fold lower expression in ‘Topaz’ than in the fully russeted ‘Patte de Loup’, respectively. *MdOSC1* and *MdOSC5* expression appeared to be differentially regulated in the fully russeted apples compared with the semi‐russeted and waxy groups. This trend was particularly marked in the fully russeted ‘Patte de Loup’ cultivar, which also displayed high level of expression of some relevant suberin‐related genes (Legay *et al*., 2015), and the waxy ‘Royal Gala’, which presented the highest *MdOSC1* expression.

No clear link could be established between *MdOSC4* and *MdOSC3* expression and the level of any triterpenes under investigation (Table 2). Positive and significant correlations were found between the expression of *MdOSC1* and the contents of UA and OA, as well as between *MdOSC5* expression and BA and triterpene‐caffeate concentrations (Table 2). It appears, therefore, that the production of UA and OA on the one hand, and BA and its caffeate derivatives on the other hand, is, at least partly, regulated at the transcriptional level and in a differential manner. Further studies, such as analyses during the developmental stages of russeted vs waxy‐skinned apple cultivars, would be needed to identify transcription factors that are involved in the fine regulation of apple triterpenic acids. Interestingly, a recent study (Mertens *et al*., 2016) reported the identification of two homologous transcription factors that distinctly direct triterpene saponin biosynthesis in *M. trunculata*.

**Table 2:** Pearson correlation coefficients between triterpene contents (reported in Table 1) and gene expression data (reported in Fig. 8) obtained from 20 apple cultivars

	Ursolic acid	Oleanolic acid	Oleanolic acid‐3‐*trans*‐caffeate	Betulinic acid	Betulinic acid‐3‐*trans*‐caffeate	Betulinic acid‐3‐*cis*‐caffeate	MdOSC1	MdOSC3	MdOSC5	MdOSC4
Ursolic acid										
Oleanolic acid	0.86**									
Oleanolic acid‐3‐*trans*‐caffeate	−0.32 ns	−0.58**								
Betulinic acid	−0.41 ns	−0.41 ns	0.65**							
Betulinic acid‐3‐*trans*‐caffeate	−0.65**	−0.68**	0.76**	0.79**						
Betulinic acid‐3‐*cis*‐caffeate	−0.64**	−0.69**	0.81**	0.82**	0.99**					
MdOSC1	**0.49** *	**0.66** **	−0.78**	−0.53*	−0.76**	−0.76**				
MdOSC3	0.30 ns	0.44 ns	−0.39*	−0.14	−0.48*	−0.45*	0.7**			
MdOSC5	−0.29 ns	−0.42 ns	**0.78** **	**0.67** **	**0.80** **	**0.81** **	−0.63**	−0.41 ns		
MdOSC4	−0.09 ns	0.16 ns	0.16 ns	0.31 ns	0.15 ns	0.19 ns	0.01 ns	0.29 ns	0.19 ns	
CYP716A175	0.19 ns	0.21 ns	−0.25 ns	0.09 ns	−0.33 ns	−0.29 ns	0.32 ns	0.78**	−0.31 ns	0.08 ns

Data significance level: **, *P *<* *0.01; *, *P *<* *0.05; ns, nonsignificant. Data in bold indicate positive correlations between a specific triterpene content and the expression of a specific gene.

A significant correlation was found between *MdOSC3* and *CYP716A175* (*r* ^2^ = 0.78; Table 2), whereas no significant links could be identified between the expression patterns of *CYP716A175* and the most determining OSC genes, *MdOSC1* and *MdOSC5*, suggesting that these are not regulated in a coordinated manner with the currently identified C‐28 oxidase. Therefore, it is likely that other cytochrome P450s are involved in the C‐28 oxidation of triterpene backbones. The recently published RNA sequencing data obtained from the skins of russeted vs waxy apple varieties (Legay *et al*., 2015) have identified new P450 candidates that will be worth investigating.

In contrast to the expression and function of *MdOSC4*, germanicol or its olean‐18‐ene derivatives have not been detected in the apple tissues under investigation and have never been reported in apple. Several hypotheses could explain this observation. First of all, germanicol and morolic acid may not accumulate in apple skin as they are quickly converted into unknown products, that is, saponins (after addition of sugar moieties), as previously described for morolic acid in *Mora excelsa* (Barton & Brooks, 1950) and *Mimosa hamata* (Singh *et al*., 2012), or hydroxycinnamoyl acid esters, as described in *Barringtonia* species (Gowri *et al*., 2009) (Ragasa *et al*., 2011), or oxidized at various carbon positions, as described in *Celastracea* species (Osorio *et al*., 2012). Numerous unknown triterpene metabolites have been detected in apple skin through LC‐MS profiling (McGhie *et al*., 2012), which could include derivatives of germanicol, but further structural elucidation work would be needed to confirm this. Another possible explanation is that germanicol plays a role in fruit growth and development, as it has been described for other triterpenes (Kemen *et al*., 2014; Moses *et al*., 2014), and is rapidly catabolized in apple skin. Further studies, including deep triterpene profiling and the production of *MdOSC4* mutants, would be needed to address this question.

### Conclusions

In this study, we identified, cloned, and characterized genes for three triterpene‐synthase‐related enzymes and determined their specificities. The transient expression of *MdOSC4* in *N. benthamiana* led to the formation of a mixture of triterpenes dominated by an oleanane‐type triterpene, putatively identified as germanicol (82%). Although the *MdOSC4* gene was expressed in the skins of all apple varieties under investigation, no germanicol or derivatives were detected at the metabolite level. Our work also describes a new lupeol synthase (*MdOSC5*) in *M*. × *domestica*. We identified a P450 gene that encodes an enzyme that hydroxylates initial triterpene scaffolds and, by coexpressing it together with the triterpene synthases, we showed that it can convert all the triterpene products to their respective C‐28 acid products (Fig. 1). By analysing the triterpene contents of 20 russeted, semi‐russeted and waxy apple cultivars, we demonstrated clear differences in their triterpene compositions. We established correlations between gene expression patterns and triterpene distributions in these cultivars. Importantly, we showed that the gene expression of *MdOSC1* and that of *MdOSC5* were coordinated and reflected at the metabolite level, providing evidence that regulation of gene expression plays an essential role in apple triterpene biosynthesis. Taken together, the results of this study allow the identification of key enzymes involved in triterpene accumulation in the apple fruit cuticle. This knowledge is an essential prerequisite for both the development of new elite apple varieties and the large‐scale *in vitro* production of specific apple triterpenes using bioengineering approaches.

## Author contributions

C.M.A. and W.A.L. planned and designed the research experiments. C.M.A., W.A.L., S.L., A.D., M.P., N.N., and C.B. contributed to molecular analyses. J.M.C., C.M.A. and A.D. carried out chemical analyses. M.L. conducted fieldwork and advised on the plant material to be used. W.A.L., Y.L., and J‐F.H. supervised part of the work. C.M.A. and W.A.L. wrote the manuscript.

## Supporting information

Please note: Wiley Blackwell are not responsible for the content or functionality of any supporting information supplied by the authors. Any queries (other than missing material) should be directed to the *New Phytologist* Central Office.

**Fig. S1** Chromatograms of typical LC‐APCI‐MS analysis of the products of MdOSC3.
**Fig. S2** Expression analysis of triterpene biosynthetic genes (*MdOSC1*,* MdOSC3*,* MdOSC4* and *MdOSC5*) by real‐time qPCR on RNA extracted from apple skin tissues.
**Fig. S3** Mass spectra of lupeol, germanicol, β‐amyrin, and α‐amyrin.
**Fig. S4** Chromatographic trace of an extract of *Nicotiana benthamiana* leaf transiently transformed with *MdOSC4* and the same sample spiked with taraxerol.
**Fig. S5** Alignment of P450 predicted amino acid sequences from apple (CYP716A175) and other species.
**Fig. S6** Mass spectral comparison (MS1, MS2, and MS3) between betulinic acid, ursolic acid, oleanolic acid, and a putative morolic acid.
**Table S1** Primer sequences and properties of triterpene‐related and housekeeping genes used in this study
**Table S2** Summary of the skin triterpene composition of 20 apple cultivars
