# Supplementary material for: Multifunctional oxidosqualene cyclases and cytochrome P450 involved in the biosynthesis of apple fruit triterpenic acids
Source: New Phytol. 2016 May 23;211(4):1279–94. doi: 10.1111/nph.13996 (PMC5089662; doi:10.1111/nph.13996)
Supplement: Supplementary file 1 — Fig. S1 Chromatograms of typical LC‐APCI‐MS analysis of the products of MdOSC3. Fig. S2 Expression analysis of triterpene biosynthetic genes (MdOSC1, MdOSC3, MdOSC4 and MdOSC5) by real‐time qPCR on RNA extracted from apple skin tissues. Fig. S3 Mass spectra of lupeol, germanicol, β‐amyrin, and α‐amyrin. Fig. S4 Chromatographic trace of an extract of Nicotiana benthamiana leaf transiently transformed with MdOSC4 and the same sample spiked with taraxerol. Fig. S5 Alignment of P450 predicted amino acid sequences from apple (CYP716A175) and other species. Fig. S6 Mass spectral comparison (MS1, MS2, and MS3) between betulinic acid, ursolic acid, oleanolic acid, and a putative morolic acid. Table S1 Primer sequences and properties of triterpene‐related and housekeeping genes used in this study Table S2 Summary of the skin triterpene composition of 20 apple cultivars [file NPH-211-1279-s001.pdf]

New Phytologist

New Phytologist Supporting Information Figs S1–S6 and Tables S1 & S2

Article title: Multifunctional oxidosqualene cyclases and cytochrome P450 involved in the biosynthesis of apple fruit triterpenic acids

Authors: Christelle M. Andre, Sylvain Legay, Amélie Deleruelle, Niels Nieuwenhuizen, Matthew Punter, Cyril Brendolise, Janine M. Cooney, Marc Lateur, Jean-François Hausman, Yvan Larondelle and William A. Laing

Article acceptance date: 29 March 2016

The following Supporting Information is available for this article:

Fig. S1 Chromatograms of typical LC-APCI-MS analysis of the products of MdOSC3.

Fig. S2 Expression analysis of triterpene biosynthetic genes (MdOSC1, MdOSC3, MdOSC4, MdOSC5) by real-time qPCR on RNA extracted from apple skin tissues.

Fig. S3 Mass spectra of lupeol, germanicol, β-amyrin, and α-amyrin.

Fig. S4 Chromatographic trace of extract of *Nicotiana benthamiana* leaf transiently transformed with MdOSC4 and the same sample spiked with taraxerol.

Fig. S5 Alignment of P450 predicted amino acid sequences from apple (CYP716A175) and other species.

Fig. S6 Mass spectra comparison (MS1, MS2, and MS3) between betulinic acid, ursolic acid, oleanolic acid, and a putative morolic acid.

Table S1 Primer sequences and properties of triterpene-related and housekeeping genes in this study.

Table S2 Summary of the skin triterpene composition of 20 apple cultivars

Table S1 Primer sequences and properties of triterpene-related and housekeeping genes used to (a) isolate oxidosqualene cyclases and (b) used on the panel of 20 apple cultivars

(a) Primers used to isolate Oxidosqualene Cyclases

Primer name	Sequence
attB1 F_OSC4_MR	GGGGACAAGTTTGTACAAAAAAGCAGGCTCCATGTGGAAGCTTAAGGTCG
attB2 R_OSC4_MR	GGGGACCACTTTGTACAAGAAAGCTGGGTCTAATCACGCTTTGGAAGG
attB1 F_OSC5_RG	GGGGACAAGTTTGTACAAAAAAGCAGGCTCCATGTGGAAGCTTAAGGTCG
attB2 R_OSC5_RG	GGGGACCACTTTGTACAAGAAAGCTGGGTTTAGACATCAACAAATGGAACCC

(b) Primers used to measure gene expression by PCR

Genes	Accession Number	Primer Sequences (5'-3')	Size (bp)	T_m (°C)	E	References
eF-1α	AJ223969.1	F: ACTGTTCCTGTTGGACGTGTTG<br>R: GAGTTGGAAGCAACGTACCC	208	82.5	1.861	Giorno *et al*. (2012)

IMPA-9	CN909679	F : TCGTGAACTCAGGCGCTTACTG<br>R : AAGCAACGGTAAAGCGGGCAAC	205	83.5	1.953	Giorno *et al*. (2012)
MdGAPDH	EB146750	F : TGAGGGCAAGCTGAAGGGTATCTT<br>R: TCAAGTCAACCACACGGGTACTGT	185	82.5	1.874	Malladi & Hirst (2010)
MdACTIN	EB127077	F: ACCATCTGCAACTCATCCGAACCT<br>R: ACAATGCTAGGGAACACGGCTCTT	185	82	1.919	Malladi & Hirst (2010)
MdOSC1	FJ032006.1	F : TTGTACTACTAATCCAGTGATCAAGATGTGG<br>R : CTCTCTTAGTATCTGAAAACGCCATAGGAG	238	84	1.857	Brendolise *et al*. (2011)
MdOSC3	FJ032008.1	F : GCAATCGTGATCAAAGAAGATGTGGAGG<br>R : TTCTCTTAAAATCTGAAAACGCCATAGG	232	84	1.882	Brendolise *et al*. (2011)
MdOSC4	KT383435	F : CTCTCGAAGTAACAATGAAGCACA	124	79	1.864	

		R : TAATCACCATTTGGGTCTTCAG			
MdOSC5	KT383436	F : TATGCATCCAGCAAAAATGTTT	152	77.5	1.851
		R : TCCAATTAATTTCGCCATAAGGT			
CYP716A175	EB148173	F : AGGCACGTTCCTCGCTTC	70	77.5	1.974
		R : CAAACCCTAAGAGGAGGGTCA			

Table S2 Summary of the skin triterpene composition of 20 apple cultivars separated into three russeting groups: russeted (four cultivars), semi-russeted (nine cultivars), and waxy (seven cultivars)

		Triterpene Acids (nmol g^{-1} DW)			Triterpene-caffeates (nmol g^{-1} DW)		
	Total Triterpenes	Ursolic acid	Oleanolic acid	Betulinic acid	Oleanolic acid -3-trans-caffeate	Betulinic acid -3-trans-caffeate	Betulinic acid -3-cis-caffeate
Russeted							
Average	6899[a]	1569[a]	800[a]	1628[b]	416[b]	2115[c]	371[c]
Min–Max	4971–8075	670–2802	500–1270	1094–2095	248–511	1287–3722	266–586
Semi-russeted							
Average	5567[a]	1981[ab]	1515[b]	1028[ab]	205[b]	693[b]	145[b]
Min–Max	4228–7636	1464–2504	991–1974	527–1712	72–406	291–1312	68–257
Waxy							
Average	5864[a]	2839[b]	2045[b]	687[a]	90[a]	159[a]	45[a]
Min	3562–7379	1680–3816	1203–2540	324–834	23–175	82–252	25–73

Data are expressed in nmol g^{-1} DW. Data were obtained after HPLC-DAD analysis as described in the Materials and Methods section. Averages with no letter in common within a column are significantly different (*P* < 0.05; One-way ANOVA, Tukey's pairwise results).

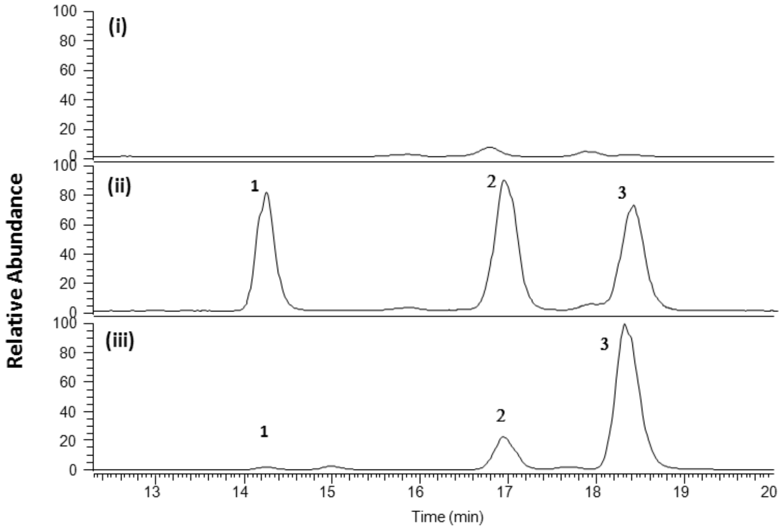

Fig. S1 Chromatograms of typical LC-APCI-MS analysis of the products of MdOSC3 (iii) after transient expression in *Nicotiana benthamiana*, following the methodology described in the Materials and Methods section. p19 was used as a negative control (i) and mixed with authentic standards at 20 µg ml^{-1} (1, lupeol; 2, β-amyrin; 3, α-amyrin) (ii). Compounds were identified and quantified on the basis of their mass spectral data (Fig. S3). Chromatograms are presented as selected ion plots of the m/z 409.8 [MH-H2O]+ ion.

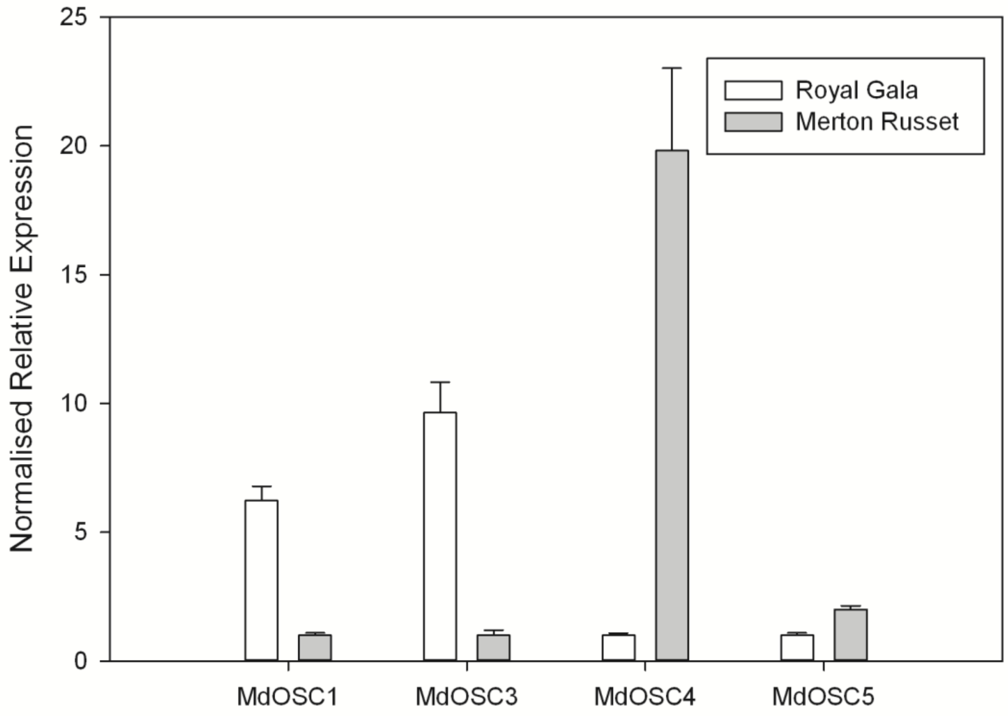

Fig. S2 Expression analysis of triterpene biosynthetic genes (MdOSC1, MdOSC3, MdOSC4, MdOSC5) by real-time qPCR on RNA extracted from apple skin tissues. Error bars show SE (n = 3).

New Phytologist

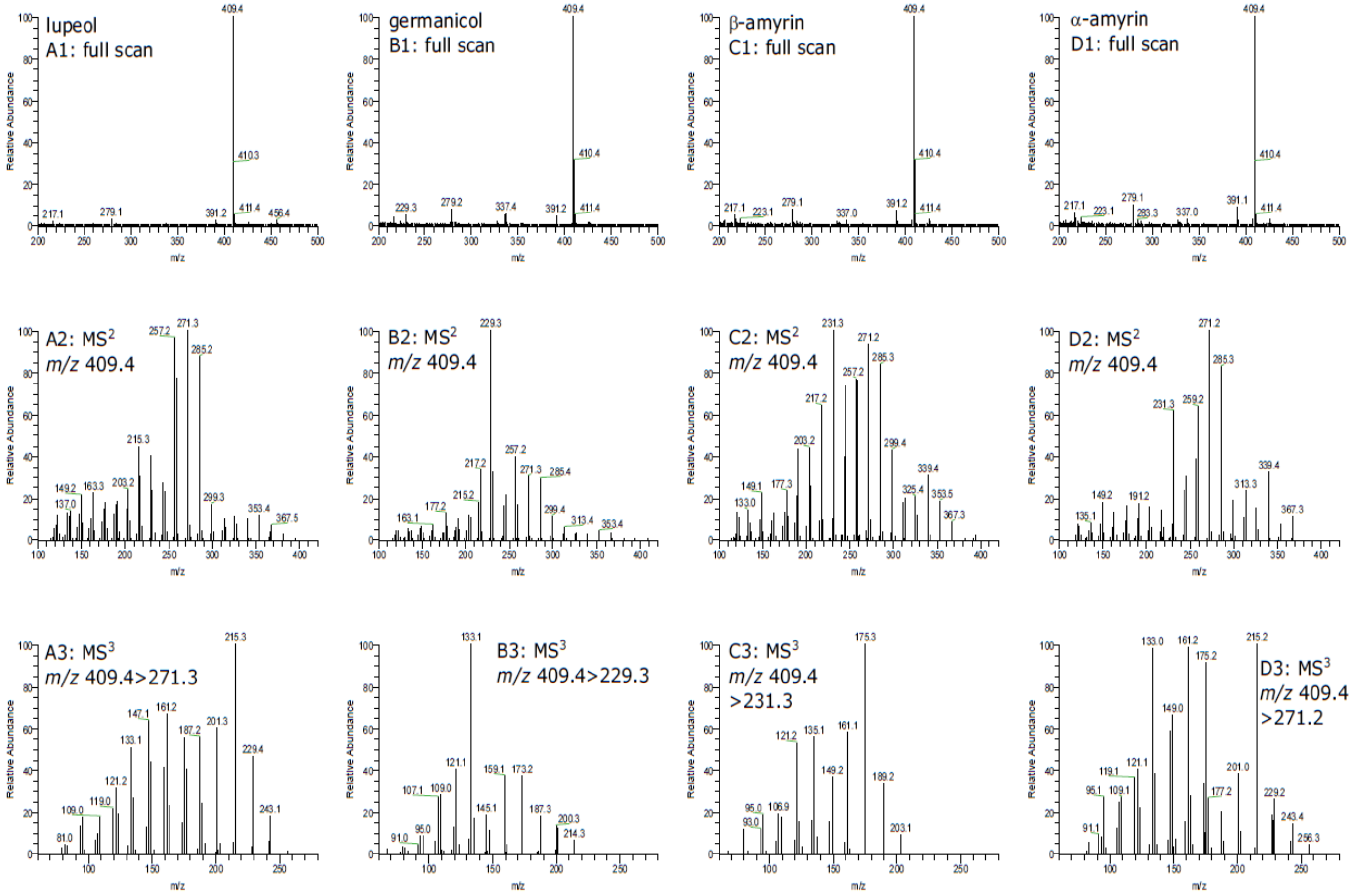

Fig. S3 Mass spectra comparison (MS1, MS2, and MS3) between lupeol, germanicol, β-amyrin, and α-amyrin.

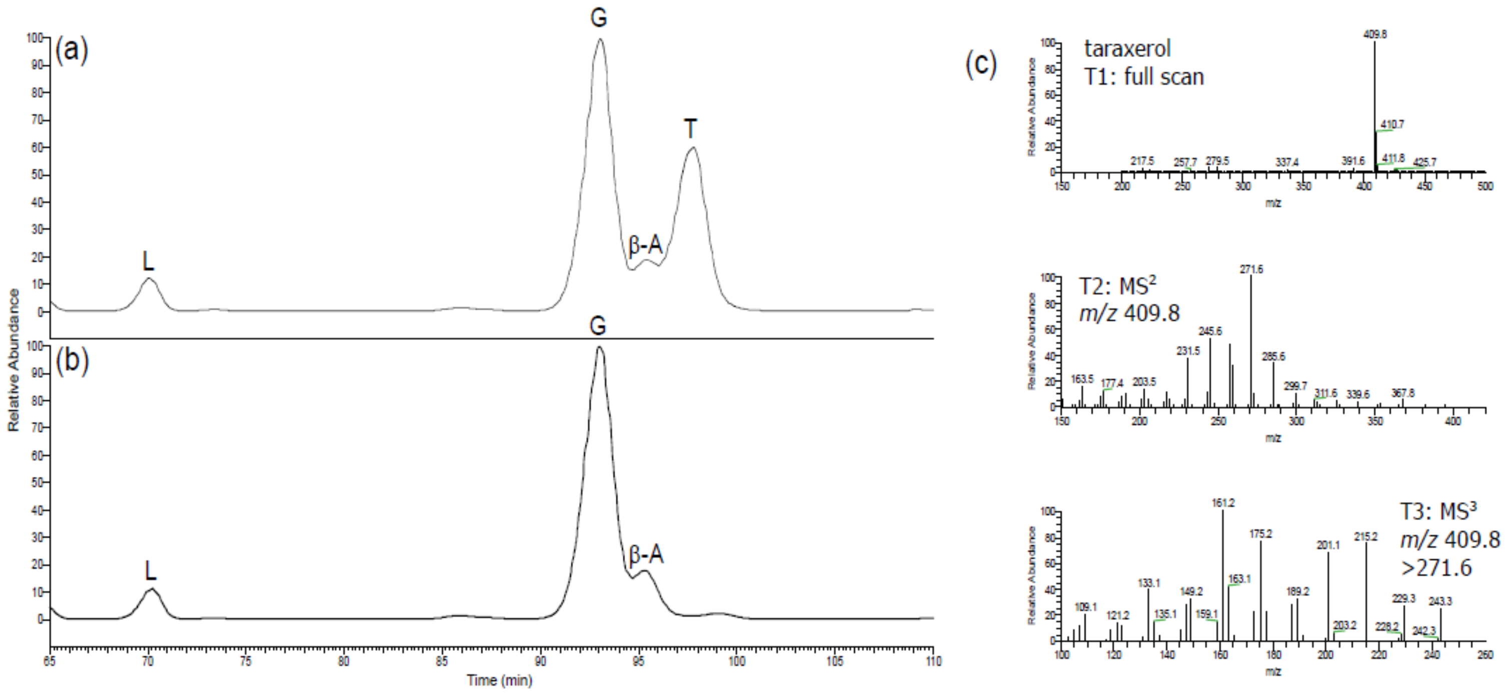

Fig. S4 Chromatographic trace of extract of *Nicotiana benthamiana* leaf transiently transformed with MdOSC4 (b) and the same sample spiked with taraxerol (T) (a) showing no taraxerol in the leaf sample. Mass spectra of the taraxerol peak (MS1, MS2, and MS3) (c). Transient expression of MdOSC4 (b) led to the formation of germanicol (G), β-amyrin (β-A), and lupeol (L). The presence of taraxerol (T) could therefore be excluded.

Fig. S5 Alignment of P450 predicted amino acid sequences from apple (CYP716A175) and other species. The prefix is the cytochrome P450 designation, and the suffix identifies the species as follows: At, *Arabidopsis thaliana*; Aa, *Artemisia annua*; Mt, *Medicago truncatula*; Vv, *Vitis vinifera*; Cp, *Carica papay*; Pt, *Populus trichocarpa*; St, *Solanum tuberosum*; Nt, *Nicotiana tobaccum*. GenBank (GB) numbers (or Arabidopsis identifiers) in the order listed in the alignment are: AT5G36140 (CYP716A2-At), AT5G36110 (CYP716A1–At), XP_002264643 (CYP716A20-Vv), XP_002280969.1 (CYP716A19-Vv), XP_002309057.1 (CYP716A8-Pt), ABC94483.1 (CYP716A14-Aa), XP_002324668.2 (CYP716A3-Pt), XP_002325241 (CYP716A6-Pt), XP_006338129 (CYP716A13-St), no GB identifier (CYP716A36-Nt), CBN88268.1 (CYP716A12-Mt), BAJ84106 (CYP716A15-Vv), BAJ84106 (CYP716A17-Vv), no GB identifier (CYP716A16-Cp). The text/background colour code refers to the degree of similarity between the different sequences: red/yellow, identical; dark blue/turquoise, conservative; black/green, block of similar; black/white, nonsimilar.

```
                  1                                                50
 CYP716A2-At     -------MYLTIIFLFISS-IIFPLLFFLGKHLSNFRYPNLPPGKIGFPL
 CYP716A1-At     ------MYMAIMIILFLSS-ILLSLLLLLRKHLSHFSYPNLPPGNTGLPL
CYP716A20-Vv     -MELLLLSFLLLMALSLS--FWIRFFVHKLEKSSG---INLPPGKMGFPF
CYP716A19-Vv     -MELSLLHILPWATLFTT--LSLSFLIYKLMIISHGTPRNLPSGNTGLPY
 CYP716A8-Pt     -MELPFISLLPYGILFIISAVSLSYLINKHKYYLS-SLNNLPPGNTGLPL
CYP716A14-Aa     MVDLILIYSIFLSLIVVLVPLSLYFVFYKSKPVVD---RKLPPGQTGWPV
 CYP716A3-Pt     --------------------------------------------------
 CYP716A6-Pt     ----MQNFFLNLLLAVFFITFFISIIVYKHRSKFK--HPNLPPGSSGLPY
CYP716A13-St     ---MEFLYVSLVCVFVFLVSLLLLYKNKS-GEG-------LPPGKTGWPV
CYP716A36-Nt     ---MEVFYLYLLCLFTLFTSLALHFIFYKRNSGSL--TGTLPPGKTGWPF
CYP716A12-Mt     --MEPNFYLSLLLLFVSFISLSLFFIFYKQKSP-----LNLPPGKMGYPI
CYP716A15-Vv     ---MEVFFLSLLLIFVLSVSIGLHLLFYKHRSHFT--GPNLPPGKIGWPM
CYP716A17-Vv     ---MEVFFLSLLLIFVLSVSIGLHLLFYKHRSHFT--GPNLPPGKIGWPM
CYP716A16-Cp     ---MEHLYLTLLLAFVSFVSLSLFIIFYKHRSPYA--YPNLPPGKIGLPF
  CYP716A175     ---MEHFYLTLLLGFVSFITLSLSVLFYRHRAQFV-GTNLPPGKVGYPV

                 51                                              100
 CYP716A2-At     IGETLSFLSAGRQGHPEKFVTDRVRHFSSG---IFKTHLFGSPFAVVTGA
 CYP716A1-At     IGESFSFLSAGRQGHPEKFITDRVRRFSSSSSCVFKTHLFGSPTAVVTGA
CYP716A20-Vv     IGESLEFLRMGRKGTPERFIQDRMAKYSTQ---IFKTCLLGEPTAVVCGA
CYP716A19-Vv     IGESIQFLSNGRKGHPEKFISERMLKFSSK---VFKTSLFGETAAVFCGS
 CYP716A8-Pt     IGESLEFLTTGQKGQPEKFILDRMAKFSSK---VFKTSLFCEPTAVFCGA
CYP716A14-Aa     IGETLEFLTNGWKGHPEKFIFDRMARFSPH---VFKTSLMLEDAAVFCGS
 CYP716A3-Pt     ----------------------------------------------MLNGV
 CYP716A6-Pt     IGETLELLLTGCKGHPQKFFLDRKAKYASE---VFKTNLFCQPAAVLCGA
CYP716A13-St     FGESLEFLSTGWKGHPEKFIFDRVAKYSSS---VFKTHLLGEEAAVFCGA
CYP716A36-Nt     IGESFEFLSTGWKGHPEKFVFDRMTKYSSS---VFKTHLLGEKAAVFCGP
CYP716A12-Mt     IGESLEFLSTGWKGHPEKFIFDRMRKYSSE---LFKTSIVGESTVVCCGA
CYP716A15-Vv     VGESLEFLSTGWKGHPEKFIFDRISKYSSE---VFKTSLLGEPAAVFAGA
CYP716A17-Vv     VGESLEFLSTGWKGHPEKFIFDRISKYSSE---VFKTSLLGEPAAVFAGA
CYP716A16-Cp     VGESLEFLSTGWKGHPEKFIFDRIARYSSV---IFKTNLLGEPAAVFCGA
  CYP716A175     IGETYQFLATGWKGHPEKFIFDRMTKYSSE---VFKTSLMGEKAAIFCGA

                 101                                             150
```

```
    CYP716A2-At   SGNKFLFTNENKLVISWWPDSVNKIFPSSTQTSSK-EEAIKTRMLLMPSM
    CYP716A1-At   SGNKFLFTNENKLVVSWWPDSVNKIFPSSMQTSSK-EEARKLRMLLSQFM
   CYP716A20-Vv   AGNKLLFSNENKLVTSWWPRSVEKIFPSSLQTSTK-EESMKTRKLLPAFL
   CYP716A19-Vv   AGNKFLFSNENKLVTAWWPSSVNKIFPSSLQTSSQ-EESKKMRKLLPGFL
    CYP716A8-Pt   AGNKFLFSNENKLVTAWWPDSVNKIFPSSQQTSSQ-EESKKMRKLFPLFF
   CYP716A14-Aa   AGNKFLFSNENKLVKAWWPASVEKILPSAKETTNQ-------RKMLSRSF
    CYP716A3-Pt   AGNKFLFSNENRLVETWWPEFVNKIFPSAVEKSPK-EEAKRMRRLFPPFL
    CYP716A6-Pt   AGNRFLFSNKNKVLKAWYPDFVCKIFPSSVQRPLI-EQVDRLRTLLPELL
   CYP716A13-St   SGNKFLFSNENKLVQAWWPNSVNKVFPSSTQTSSK-EEAIKMRKMLPNFF
   CYP716A36-Nt   TCNKFLFSNENKLVQAWWPDSVNKVFPSSTQTSSK-EEAIKMRKMLPNFF
   CYP716A12-Mt   ASNKFLFSNENKLVTAWWPDSVNKIFPTTSLDSNLKEESIKMRKLLPQFF
   CYP716A15-Vv   AGNKFLFSNENKLVHAWWPSSVDKVFPSSTQTSSK-EEAKKMRKLLPQFF
   CYP716A17-Vv   AGNKFLFSNENKLVHAWWPSSVDKVFPSSTQTSSK-EEAKKMRKLLPQFF
   CYP716A16-Cp   ACNKFLFSNENKLVTAWWPNSVNKIFPTSLQTSSI-EESKKMRKLLPQFL
     CYP716A175   ACNKFLFSNENKLVTAWWPSSVNKVFPSSLETSAK-EEAKKMRKMLPNFM

                  151                                            200
    CYP716A2-At   KPEALRRYVGVMDEIAQKHFETEWANQDQLIVFPLTKKFTFSIACRLFLS
    CYP716A1-At   KPEALRRYVGVMDEIAQRHFETEWANQDQVIVFPLTKKFTFSIACRSFLS
   CYP716A20-Vv   KPEALQKYVGIMDSIAKWHLDNHWDLNETVTVFPLAKQYTFMVACRLFLS
   CYP716A19-Vv   KPEALQRYISIMDVIAQRHFESSWNNKEEVTVFPLAKMFTFWLACRLFLS
    CYP716A8-Pt   KPESLQRYISVMDVIAQRHLASDWEGKQEVSVFPLAKTYTFWLACRLFLS
   CYP716A14-Aa   RPESLRQYVPVMDMMAQRHFKTEWDGMDQIVTHEVTQNFTFSLACKIFVS
    CYP716A3-Pt   KPEALRRYIGTMDMVTKRHFALEWGNKAEVVVFPLAKSYTFELACRLFLS
    CYP716A6-Pt   RPDALKRYVGIFDKAAGRHFASEWENKKVVVVFPLAKRFTFGLACSLFLS
   CYP716A13-St   KPEALQRYVGIMDHITQRHFASGWENKEQVVVFPLTKRYTFWLACRLFLS
   CYP716A36-Nt   KPEALQRYVGIMDHIAQRHFASSWENKNQIEVFPLAKRYTFWLACRLFVS
   CYP716A12-Mt   KPEALQRYVGVMDVIAQRHFVTHWDNKNEITVYPLAKRYTFLLACRLFMS
   CYP716A15-Vv   KPEALQRYIGIMDHIAQRHFADSWDNRDEVIVFPLAKRFTFWLACRLFMS
   CYP716A17-Vv   KPEALQRYIGIMDHIAQRHFADSWDNRDEVIVFPLAKRFTFWLACRLFMS
   CYP716A16-Cp   KPEALQRYIGIMDGIAQRHFESGWENKEEVKVFPLAKSYTFWIACRLFMS
     CYP716A175   KPEALQRYIGIMDTVARRHFAEGWENKKEVEVFPLAKNYTFWLAARLFVS

                  201                                            250
    CYP716A2-At   MDDLERVRKLEEPFTTVMTGVFSIPIDLPGTRFNRAIKASRLLSKEVSTI
    CYP716A1-At   MEDPARVRQLEEQFNTVAVGIFSIPIDLPGTRFNRAIKASRLLRKEVSAI
   CYP716A20-Vv   IDDPKHIAKFANPFHILAAGVMSIPINFPGTPFNRAIKAADSVRKELRAI
   CYP716A19-Vv   VEDPDHVEKLAEPFNELAAGIIALPIDLPGTSFNKGIKASNLVRKELHAI
    CYP716A8-Pt   MEDPEEVQKFAKPFNDLAAGIISIPIDLPWTPFNRGVKASNVVHKELLKI
   CYP716A14-Aa   IEDPEEVKHLSGPFEKFAPGIFSIPIDLPWTPLRRAIHAGNFIRKEIIAI
    CYP716A3-Pt   IEDPSHIARFSHPFNHITSGIFTIPIAFPGTPFNRAIKATKLIRIELLAI
    CYP716A6-Pt   IEDPDHIAKLASPFNLVVSGIFSIPIDLPGTPLSRAIKASTIIRTELFAI
   CYP716A13-St   VEDPNHVAKFADPFDVLASGLISIPIDLPGTPFNRAIKASNFIRKELVRI
   CYP716A36-Nt   VEDPNHVAKFADPFNVLASGLISIPIDLPGTPFNRAIKASNLIRKELLLI
   CYP716A12-Mt   VEDENHVAKFSDPFQLIAAGIISLPIDLPGTPFNKAIKASNFIRKELIKI
   CYP716A15-Vv   IEDPAHVAKFEKPFHVLASGLITVPIDLPGTPFHRAIKASNFIRKELRAI
   CYP716A17-Vv   IEDPAHVAKFEKPFHVLASGLITVPIDLPGTPFHRAIKASNFIRKELRAI
   CYP716A16-Cp   IEDPNHVERFAEPFHHLASGVISIPIDLPGTAFNRGIKASNFIRKELLKI
     CYP716A175   LEDSVEIAKLGDPFAVLASGIISMPLDFPGTPFYKAIKASNFIREELTKI

                  251                                            300
    CYP716A2-At   IRQRKEELKAGKVSVE-QDILSHMLMNIGET----KDEDLADKIIALLIG
    CYP716A1-At   VRQRKEELKAGKALEE-HDILSHMLMNIGET----KDEDLADKIIGLLIG
   CYP716A20-Vv   IKQRKIQVLAGKSSSSKHDILSHMLTTTDENGQFLNEMDIADKILGLLIG
   CYP716A19-Vv   IKKRKMNLADNKASTT-QDILSHMLLTCDENGEYMNEEDIADKILGLLVG
    CYP716A8-Pt   IKQRKIDLAENKASPT-QDILSHMLTTADDNGQCMKKIDIADKILGLLVG
   CYP716A14-Aa   IKQRKIDLADGKASPT-QDILSQMLCDEESQN--IAEADTADVIIGLLIG
    CYP716A3-Pt   IRQRKKDLAEGKASPT-QDILSHMLLSNDANGQYMNEVEIADKIIALLLG
    CYP716A6-Pt   IKQRKKDLAKGKASPK-QDILSHMLAC-DEKGAFMSELDIADTILALLAS
   CYP716A13-St   IKQRKIDLGEGKASST-QDILSHMLLTCDENGKFMGDLDIADKILGLLIG
   CYP716A36-Nt   IKQRKVDLAEGKASPT-QDILSHMLLTSDENGKYMHELDIADKILGLLIG
   CYP716A12-Mt   IKQRRIDLAEGTASPT-QDILSHMLLTSDENGKSMNELNIADKILGLLIG
   CYP716A15-Vv   IKQRKIDLAEGKASQN-QDILSHMLLATDEDGCHMNEMEIADKILGLLIG
   CYP716A17-Vv   IKQRKIDLAEGKASQN-QDILSHMLLATDEDGCHMNEMEIADKILGLLIG
   CYP716A16-Cp   IKQRKIDLGNGSASPT-QDILSHMLLTSDESGQFMSELDIADKILGLLIG
     CYP716A175   IKQRKIDLAEGKASPT-QDILSHMLLLCDEHGSHMKEHDIADKILGLLIG

                  301                                            350
```

```
  CYP716A2-At    GHDTTSIVCTFVVNYLAEFPHIYQRVLEEQKEILNNKDVN-EKLTWEDIE
  CYP716A1-At    GHDTASIVCTFVVNYLAEFPHVYQRVLQEQKEILKEKKEK-EGLRWEDIE
 CYP716A20-Vv    GHDTASAVITFIIKYLAELPQVYNEVLKEQMEVAAGKKSG-EMLDWEDIQ
 CYP716A19-Vv    GHDTASATITFIVKFLAELPHVYDEVFKEQMEIAKSKAPG-ELLNWEDIP
  CYP716A8-Pt    GHDTASAAITFIVKYLAELPHVYNKLLEEQREIAKTKTPG-ELLNWEDIQ
 CYP716A14-Aa    GHDNASSTCAFIVKFLADLPEIYEGVLKEQLEIAKFKAPG-ELLNWEDLS
  CYP716A3-Pt    AHDSTGTACTFIVKYLAEMPHIYEAVYKEQAEIIKSKAPG-ELLNWVDIQ
  CYP716A6-Pt    AHESTSAACAFIVKYLAELPLIYNAVYKEQMKISETKAPGDDLLNWNDIQ
 CYP716A13-St    GHDTASSACAFIVKYLAELPHIYQRVYTEQMEIAKSKGPG-ELLRWEDIQ
 CYP716A36-Nt    GHDTASSACTFILKYLAEFPEIYEGVYKEQMEIAMSKSPG-ELLNWDDIQ
 CYP716A12-Mt    GHDTASVACTFLVKYLGELPHIYDKVYQEQMEIAKSKPAG-ELLNWDDLK
 CYP716A15-Vv    GHDTASAAITFLIKYMAELPHIYEKVYEEQMEIANSKAPG-ELLNWDDVQ
 CYP716A17-Vv    GHDTASAAITFLIKYMAELPHIYEKVYEEQMEIANSKAPG-ELLNWDDVQ
 CYP716A16-Cp    GHDTASAACTFIVKYLAELPHIYQKVYEEQMEIAKSKAAG-EMLNWDDIQ
   CYP716A175    GHDTASATCTFIVKYLAELPHIYDEVYKEQMEVLSAKAPG-DLLNWDDLQ

                 351                                            400
  CYP716A2-At    KMRYSWNVACEVMRIVPPLAGTFREAIDHFSFKGFYIPKGWK-LYWSATA
  CYP716A1-At    KMRYSWNVACEVMRIVPPLSGTFREAIDHFSFKGFYIPKGWK-LYWSATA
 CYP716A20-Vv    KMKYSWNVANEVMRLAPPLQGSFREAITDFTYAGFSIPKGWKFLYWSTNA
 CYP716A19-Vv    KMRYSWNVACEVMRLAPPVQGAFREAMNDFIFEGFSIPKGWK-LYWSTHS
  CYP716A8-Pt    RMRYSWNVACEVMRVAPPLQGAFREAMTEFNYAGFTIPKGWK-LYWSANT
 CYP716A14-Aa    KMKYSWNVACEVLRLAPPLQGSFREAMTDFVYNGYSIPKGWK-LYWSALS
  CYP716A3-Pt    KMKYSWNVACETLRLSPPFIGNFKEAIKDFTFNGFAIPKGWKASHFLTLY
  CYP716A6-Pt    NMTYSWNVIREVLRLCPTFP-NVREAIHDFDFNGFSIPKGWKAKYFP---
 CYP716A13-St    KMKYSWNVACEVLRLAPPLQGAFREALSDFIFNGFSIPKGWK-IYWSANS
 CYP716A36-Nt    KMKYSWNVACEVLRLASPLQGAFREAINDFIFNGFYIPKGWK-LYWSANS
 CYP716A12-Mt    KMKYSWNVACEVMRLSPPLQGGFREAITDFMFNGFSIPKGWK-LYWSANS
 CYP716A15-Vv    NMRYSWNVACEVMRLAPPLQGAFREAITDFVFNGFSIPKGWK-LYWSANS
 CYP716A17-Vv    KMRYSWNVACEVMRLAPPLQGAFREAITDFVFNGFSIPKGWK-LYWSANS
 CYP716A16-Cp    KMKYSWNVACEVLRLAPPLQGAFRQALYDFIFDGFSIPKGWK-LYWSANS
   CYP716A175    KMKYSWNVAQEVLRLAPPLQGAFREALSDFVFNGFTIPKGWK-LYWSANS

                 401                                            450
  CYP716A2-At    THK-----NPEYFPEPEKFEPSRFEGSGPKPYTYVPFGGGSRICPGREYA
  CYP716A1-At    THM-----NPDYFPEPERFEPNRFEGSGPKPYTYVPFGGGPRMCPGKEYA
 CYP716A20-Vv    THK-----NPDYFPDPEKFDPSRFEGNGPIPYTYVPFGGGPRMCPGKEYA
 CYP716A19-Vv    THR-----NPEFFPKPEKFDPSRFDGKGPAPYTYVPFGGGPRMCPGKEYA
  CYP716A8-Pt    THK-----NPECFPEPENFDPSRFEGNGPAPYTFVPFGGGPRMCPGKEYA
 CYP716A14-Aa    THK-----NPEVFTEPQKLDPSRFDGKGPAPYTFVPFGGGPHMCPGREYA
  CYP716A3-Pt    WSASSTHKNPEYFSEPEKFDPSRFEGKGPAPYTFIPFGGGPRMCPGNEYA
  CYP716A6-Pt    ---------------EPERFDPSRFEGTGPAPYTFVPFGGGPMMCPGQGFA
 CYP716A13-St    THK-----SGEFFPDPEKFDPSRFEGSGPAPYTFVPFGGGPRMCPGKEYA
 CYP716A36-Nt    THK-----NPEYFPEPQKFDPSRFEGSGPAPYTFVPFGGGPRMCPGKEYA
 CYP716A12-Mt    THK-----NAECFPMPEKFDPTRFEGNGPAPYTFVPFGGGPRMCPGKEYA
 CYP716A15-Vv    THK-----SPECFPQPENFDPTRFEGNGPAPYTFVPFGGGPRMCPGKEYA
 CYP716A17-Vv    THK-----SPECFPQPENFDPTRFEGDGPAPYTFVPFGGGPRMCPGKEYA
 CYP716A16-Cp    THK-----DPECFPEPEKFDPTRFEGNGPAPYTFVPFGGGPRMCPGKEYA
   CYP716A175    THK-----NAAYFPEPFKFDPTRFEGNGPAPYTFVPFGGGPRMCPGKEYA

                 451                                                  502
  CYP716A2-At    RLEILIFMHNLVKRFKWEKVFPKENKLVADPAPIPAKGLPI-----------
  CYP716A1-At    RLEILIFMHNLVNRFKWEKVFPNENKIVVDPLPIPDKGLPI-----------
 CYP716A20-Vv    RLEILVFIHNVVRRFSWYKLHPNE-DVIVDPMPMPAKGLPIRLRHH------
 CYP716A19-Vv    RLEVLVFMHNLVRRFKWEKLLPDE-KIIVDPMPIPAKGLPIRLHHHQP----
  CYP716A8-Pt    RLEILVFLHNLVKKFRWEKLLPKE-RIIVDPMPIPSKGLPIRLHPHEAA---
 CYP716A14-Aa    RLEILVFMHHLVIKYKWEKVIPNE-QIIVNPMPKLAKGLPLRLYPRYA----
  CYP716A3-Pt    RLEILVFMHNLVKRFKFERLVLDE-KIVFDPTPKPERGLPVRLLPHKA----
  CYP716A6-Pt    RLEMLIFMHNLVKRFKFDKFVAEE-KIMFSPMPIPEKGVPIRLFPHRP----
 CYP716A13-St    RLEILVFMHHLVKRFKFEKIIPDE-KIIVNPMPIPAKGLPVRLYPHHHKP--
 CYP716A36-Nt    RLEILVFMHHLVKRFKWEKVIPNE-KIVVDPMPIPAKGLPVRLYPHKA----
 CYP716A12-Mt    RLEILVFMHNLVKRFKWEKVIPDE-KIIVDPFPIPAKDLPIRLYPHKA----
 CYP716A15-Vv    RLEILVFMHNVVKRFKWDKLLPDE-KIIVDPMPMPAKGLPVRLHPHKP----
 CYP716A17-Vv    RLEILVFMHNVVKRFKWDKLLPDE-KIIVDPMPMPAKGLPVRLHPRKP----
 CYP716A16-Cp    RLEILVFMHNVIKRFKWEKVLPDE-KIIVDPMPVPAKGLPVRLYPHAHAL--
   CYP716A175    RLEILVFMHNLVKRFKWEKVLPDE-QIVVDPLPMPAKGLPVRLFPHPKTATT
```

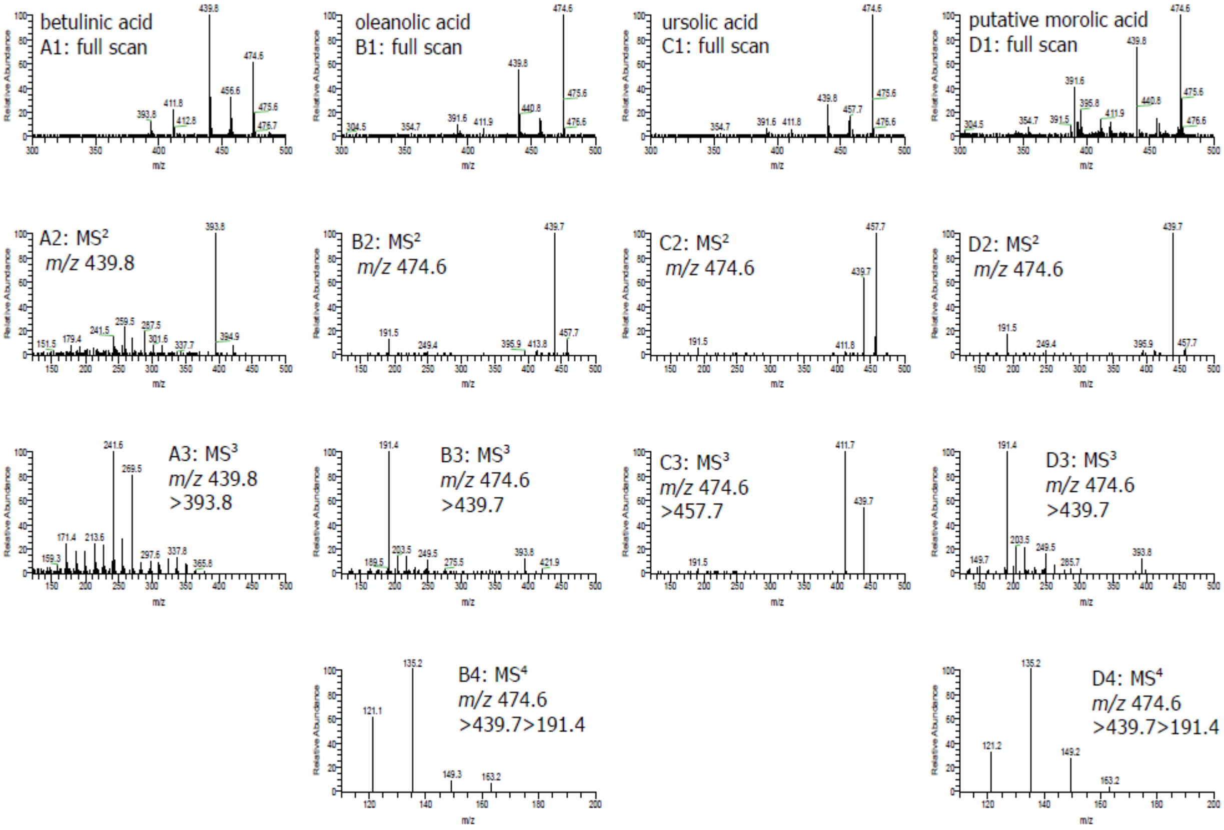

Fig. S6 Mass spectra comparison (MS1, MS2, and MS3) between betulinic acid, ursolic acid, oleanolic acid, and a putative morolic acid.

New Phytologist

References

Brendolise C, Yauk Y-K, Eberhard ED, Wang M, Chagne D, Andre C, Greenwood DR, Beuning LL. 2011. An unusual plant triterpene synthase with predominant α-amyrin-producing activity identified by characterizing oxidosqualene cyclases from *Malus* × *domestica*. *FEBS Journal* **278:** 2485–2499.

Giorno F, Guerriero G, Baric S, Mariani C. 2012. Heat shock transcriptional factors in *Malus domestica*: identification, classification and expression analysis. *BMC Genomics* **13:** 639.

Malladi A, Hirst PM. 2010. Increase in fruit size of a spontaneous mutant of “Gala”apple (*Malus*× *domestica* Borkh.) is facilitated by altered cell production and enhanced cell size. *Journal of Experimental Botany* **61:** 3003–3013.
